# GRIN lens implantation strategies for in vivo calcium imaging using miniature microscopy

**DOI:** 10.1371/journal.pone.0323256

**Published:** 2025-05-12

**Authors:** Pingping Zhao, Daniel Aharoni, Peyman Golshani

**Affiliations:** 1 Department of Neurology, David Geffen School of Medicine, University of California, Los Angeles, Los Angeles, California, United States of America; 2 West Los Angeles Veteran Affairs Medical Center, Los Angeles, California, United States of America; 3 Intellectual and Developmental Disabilities Research Center, University of California, Los Angeles, Los Angeles, California, United States of America; University of Science and Technology of China, CHINA

## Abstract

Miniature microscopy (Miniscope) has become one of the most popular and valuable neuroscience tools in the last decade. Miniscope in vivo calcium imaging during freely moving behavior has led to a number of transformative discoveries about neural coding across a large range of behaviors. The UCLA Miniscope Project is an open-source miniaturized microscopy platform that has greatly benefited the neuroscience community and led to the release of a number of different miniaturized microscopes with extended capabilities. While researchers can record from essentially any brain region through a cranial window or a gradient index of refraction (GRIN) lens, there is still a need for comprehensive protocols which describe detailed surgical procedures for successful miniaturized microscopy applications across different brain regions. Here, we provide step-by-step surgical procedures for implantation of GRIN lenses to record from a number of different brain regions including subregions in the medial prefrontal cortex (PrL, IL, DP), subregions in the hippocampus (dCA1, CA2 and vCA1), and the ventral striatum (nucleus accumbens, NAc). Moreover, we also provide surgical methods of new multi-brain regions imaging techniques developed by our group to record bilateral medial prefrontal cortex (mPFCs) or simultaneously record mPFC and NAc. Taken together, this protocol details easy and reproducible techniques for GRIN lens implantation and miniaturized microscopy in multiple structures.

## Introduction

Miniature microscopy enables recording neural activity during free behavior and allows researchers to track the same neural population over weeks to months [1–5]. Over the last decade, the UCLA Miniscope Project has provided multiple open-source miniature microscopes including the V3 Miniscope [1,4,5] and V4 Miniscope [5], the wire-free Miniscope [1] for unrestrained recordings in large environments, the E-Scope [6] for simultaneously recording calcium activity and extracellular electrical activity, and MiniFLOV [7] and MiniXL [8] for large-field-of-view recordings. The project has also provided software for data acquisition and data analysis pipelines including the Miniscope Analysis (Minian) package [9].

Yet, the effective use of any miniaturized microscopes requires successful surgical implantation of lenses or cranial windows. Successful surgeries are critical for maintaining the animals’ health for robust behavioral performance, making processing and analysis such as motion correction and cell registration significantly easier, thus leading to higher number of neurons recorded per animal. Generally, performing in vivo miniscope imaging in superficial cortical regions requires a cranial window identical to those used for table-top two photon imaging, which will not be further discussed in this paper [10–12]. For deeper subcortical regions, miniscope imaging requires a relay GRIN lens system [5,13,14]. Here, we describe surgical procedures for GRIN lens implantation for deep brain region imaging in the mouse. We will also describe how to choose the proper GRIN lens with the correct specifications and how to decide the ideal surgical approach for the targeted area. Finally, we will introduce surgical methods for simultaneous imaging of multiple brain regions with a large field-of-view (FOV) miniscope system.

## Materials and methods

The protocol described in this peer-reviewed article is published on protocols.io (https://doi.org//10.17504/protocols.io.ewov12jyogr2/v1) and is included for printing as supporting information (S1 File) with this article.

### Animals

Animal background, age, and sex should be decided according to the experimental aim. The current miniaturized microscopes are appropriate for adult (> 6 weeks old) mice. All mice were maintained on a 12h:12h light/dark cycle with food and water ad libitum. Mice were single housed for three to four weeks before *in vivo* calcium imaging and behavioral experiments.

For surgeries involved in this study, mice were anesthetized with 3–5% isoflurane-oxygen mixture and placed into a stereotactic frame (David Kopf Instruments). Then 1–2% isoflurane-oxygen mixture was used throughout the surgery. Dexamethasone (0.2 mg/kg) and lidocaine (2%) were administered 30 minutes before the start of surgery. During surgery, 1 ml 0.9% saline was injected to prevent dehydration. The ophthalmic ointment will remain on the eyes until the animal is awake and ambulatory. After surgery, mice were placed on a heating pad to maintain core temperature until it recovered from anesthesia. Post-operative care included administering Carprofen (5 mg/kg) injected subcutaneously every 12–24 hours for 48 hours along with Dexamethasone (0.2 mg/kg). Additional doses may be administered after the initial 48-hour period as needed for pain. Amoxicillin (0.25 ml/mL) was given in drinking water for 7 days. After the 7-day period, it was replaced with normal water.

Animal health and behavior were monitored by experimenter daily especially for any sign of infection or discomfort until they were euthanized. Given the presence of any untreatable health issue, surgery related infection (which may affect experimental result) or GRIN lens damage (imaging will not be performed), on the same day, the animal would be deeply anesthetized and euthanized via perfusion, or cervical dislocation (if histology is not needed). In this study, no animal died before meeting criteria for euthanasia. In total, 12 mice were used and euthanized via perfusion.

### Ethics statement

All experimental protocols were approved by the Chancellor’s Animal Research Committee of the University of California, Los Angeles, in accordance with the NIH guidelines. All researchers must obtain Animal Research Committee certification (including CITI program training and species-specific training) prior to initiating work with laboratory animals.

### Calcium indicators

There are two main methods of expressing genetically encoded calcium indicators in mice: 1. Virus injection: there are large varieties of viral constructs for cell type or projection specific expression of genetically encoded calcium indicator (GECI), GCaMPs, and we will not discuss these in detail in this paper. We refer the reader to these papers as reference for choosing the correct virus or viral combinations [15–18]. It is important to consider the titer of the virus as dilution of the virus may be necessary for adjusting the density of labeling and preventing neuronal toxicity [19]. Furthermore, prolonged viral expression may also lead to toxicity and experiments may need to be designed such that overexpression of the viral constructs does not occur over time. 2. Transgenic mouse lines: There are a number of transgenic mouse lines with expression of GCaMPs in different brain regions and with different densities [20,21]. Since the expression level is lower in transgenic animals, it is important to test miniscope imaging in the specific brain region to determine whether expression levels are sufficient for miniscope recordings. In general, transgenic lines allow recordings across a longer time span (even many months) compared to experiments using viral expression which could be limited to a few weeks.

### GRIN lens selection

The choice of relay GRIN lens includes selecting the appropriate length and diameter: 1. GRIN Lens length: GRIN lens should be long enough to reach the target area and leave extra length outside the skull (>2 mm would be ideal) such that the lens can be held in place for implantation. However, If the selected lens is too long, the installation of the baseplate will be more difficult because the experimenter will need to fill a larger gap between the baseplate and the skull with dental cement. We suggest that the lens left outside the skull is shorter than 4 mm. 2. GRIN lens diameter: Imaging through a larger diameter relay GRIN lens usually enables recording from a larger field of view and therefore larger number of neurons compared to imaging through a thinner GRIN lens. However, using larger diameter lenses will be more invasive and have the potential for perturbing the behavior and brain dynamics being studied. For any GRIN lens implantation, it is critical to first determine whether the behavior studied is impacted by the lesion created by the lens. For the novel multiple GRIN lenses imaging strategy discussed later, thinner lenses are preferred. In this case, the researcher should choose lenses whose tops are at approximately the same height post-implantation so that both regions can be in focus under the large field of view miniscope. This may require minor adjustments to the dorsal/ventral coordinates of the implantation. Below, we list the suggested lens selection for a series of brain regions according to our own experience and published studies (Tables 1 and 2):

**Table 1:** Specifications of commercial GRIN lenses.

Lens Length (mm)	Lens Diameter (mm)	Part Number
4.3	1.8	Edmund #64–531
4	1	Inscopix 1050–004595
7.3	0.6	Inscopix 1050–004597
6.1	0.5	Inscopix 1050–004599
8.4	0.5	Inscopix 1050–004600

**Table 2:** Suggested GRIN lenses for various brain region imaging.

Brain region	Selected lens (Diameter/Length mm)
dCA1	1.8/4.3 (Edmund for UCLA Miniscope V3) 1/4 (Inscopix for UCLA Miniscope V4)
CA2	1/4 (Inscopix)
vCA1	0.5/6.1 (Inscopix)
PL	1/4 (Inscopix)
IL	0.5/6.1 (Inscopix)
DP	0.5/6.1 (Inscopix)
NAc	0.6/7.3 (Inscopix)
VTA	0.6/7.3 (Inscopix)
VMH	0.5/8.4 (Inscopix)
PFC + PFC	1/4 + 1/4 (Inscopix)
PFC + NAc	0.5/6.1 + 0.5/8.4 or 1/4 + 0.5/6.1 (Inscopix)

### GRIN lens holder

**Fig 1:**
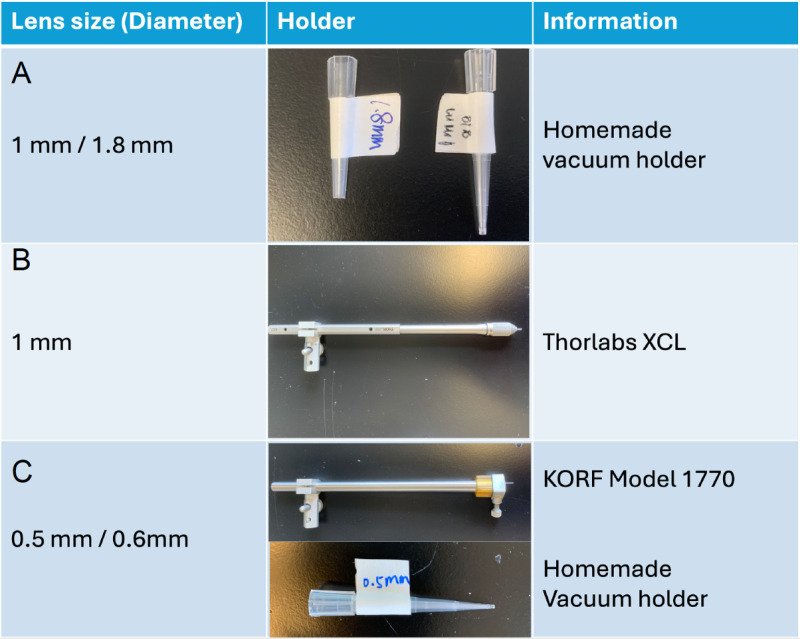
Lens holders for different sizes of GRIN lenses. **(A)** Homemade vacuum lens holders for GRIN lenses with 1 mm and 1.8 mm diameters. **(B)** Commercial lens holders for GRIN lenses with 1 mm diameters. **(C)** Lens holders for GRIN lenses with 0.5 mm and 0.6 mm diameters.

**Fig 2:**
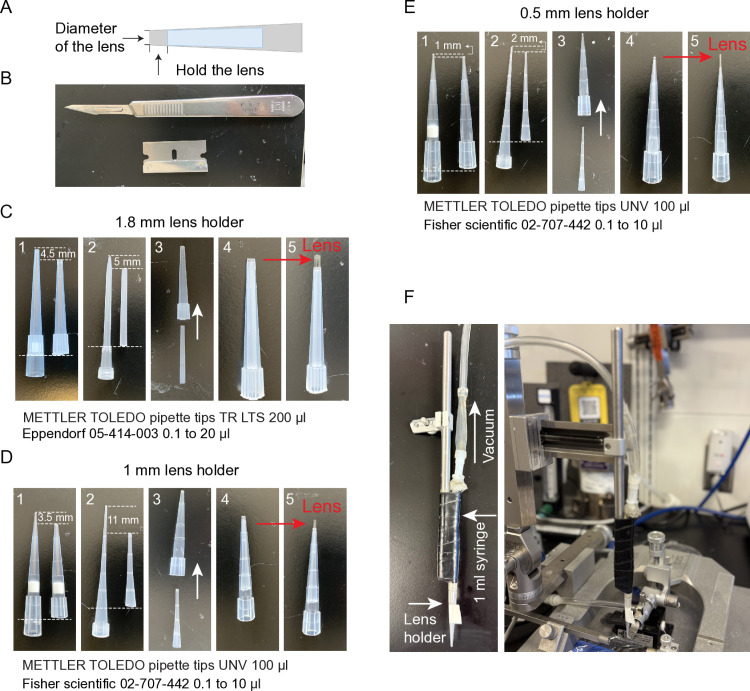
Instructions for homemade lens holders. **(A)** Diagram of homemade lens holder using two pipette tips. **(B)** Tools for making lens holders. **(C)** Instruction for making a 1.8 mm lens holder. 1, Cut 4.5 mm from the tip of METTLER TOLEDO 200 μl pipette tip and remove the filter. Cut the back of the same pipette tip to make it fit 1 ml syringe. 2, Cut 5 mm from the tip of Eppendorf 20 μl pipette tip and cut the back at where the diameter significantly changes. 3 and 4, Insert the thinner cut tip into the thicker one and push it to the end. 5, Test. **(D)** Instruction for making a 1 mm lens holder. 1, Cut 3.5 mm from the tip of METTLER TOLEDO 100 μl pipette tip and remove the filter. Cut the back of the same pipette tip to make it fit 1 ml syringe. 2, Cut 11 mm from the tip of Fisher scientific 10 μl pipette tip and cut the back approximately at where the diameter significantly changes. 3 and 4, Insert the thinner cut tip into the thicker one and push it to the end. 5, Test. **(E)** Instruction for making a 0.5 mm lens holder. 1, Cut 1 mm from the tip of METTLER TOLEDO 100 μl pipette tip and remove the filter. Cut the back of the same pipette tip to make it fit 1 ml syringe. 2, Cut 2 mm from the tip of Fisher scientific 10 μl pipette tip and cut the back approximately at where the diameter significantly changes. 3 and 4, Insert the thinner cut tip into the thicker one and push it to the end. 5, Test. **(F)** Example of the vacuum holder system.

Lens holders can be built in the lab and connected to vacuum to hold the GRIN lens using two plastic pipette tips (Figs 1 and 2). Using a vacuum holder allows the GRIN lens to be securely held during implantation but also released without any additional mechanical force when removing the holder. The GRIN lens is stabilized by the length difference between two tips. Proper calibers of the cut pipettes can tightly hold the lens and ensure proper lens orientation (make the lens vertical to the horizontal plane) prior to insertion. These holders are especially suitable for 1.8 mm and 1 mm diameter GRIN lenses.

In addition, for lenses with a thinner diameter (0.5 mm, 0.6 mm in diameter), as well as 1 mm GRIN lenses, commercial lens holders are recommended in most cases. Selections of these holders are listed in Figs 1 and 2

### Aspiration system

**Fig 3:**
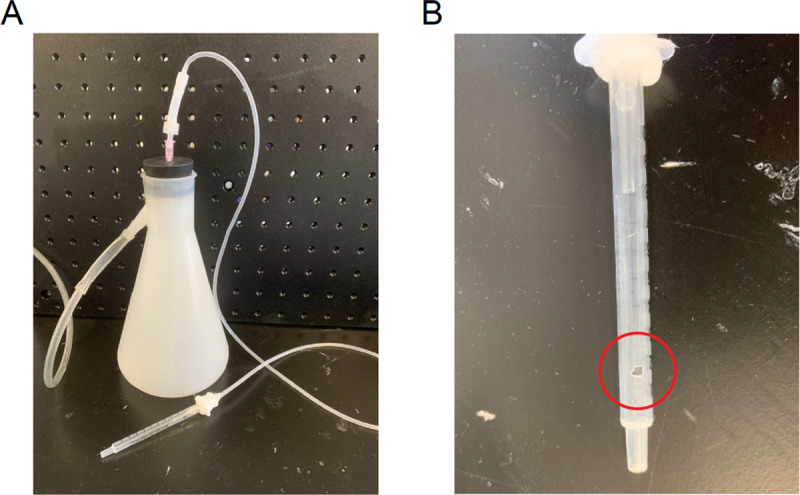
Aspiration system. (A) Aspiration system is composed of a flask, the wall vacuum port, venous infusion needle and a 1 ml syringe. (B) Drill a hole on the side of the syringe for controlling vacuum by finger pressing.

For relatively superficial subcortical brain areas, such as dCA1, ACC, dmPFC, etc, larger diameter lenses are ideal to record large neural population. To prevent the pressure from accumulated tissue under the lens, removing the tissue by aspiration is highly suggested. For implantations requiring aspiration, experimenters need to set up a vacuum system which includes tubes, a flask acting as a liquid trap and a syringe with a hole in its body to control the vacuum (Fig 3).

### Surgical instruments and other supplies and reagents

Surgical instruments are listed below (Table 3).

**Table 3:** Surgical instruments information.

Surgical instrument item	Information
Small animal stereotaxic instruments	KOPF Model 963
Mouse gas anesthesia head holder	KOPF 923B
Zygoma ear cups	KOPF 921
Surgical microscope	ZEISS Stemi 508
Isoflurane setup	SurgiVet
Homeothermic monitoring system	Harvard Apparatus
Light	Zeiss
Drill	MK-dent, HE17 Eco Line
Virus injection instrument	Drummond, NANOJECT III
FST tools	FST 14090–09; FST 10003–12; FST 11223–20; FST 11295–10

Other supplies and reagents are listed below (Table 4):

**Table 4:** Supplies and reagents for lens implantation surgery.

Item	Information
00-96 x 1/16 screw	Plastics One
27G needle	CMLsupply
30G needle	CMLsupply
31G needle	BD Veo U-100 syringes
Needle holder	KORF Model 1766
Marker pen	Sakura Pigma Micron pen 005
Cyanoacrylate glue	Krazy glue
Silicone Elastomer	Kwik-Sil (KWIK-SIL), World Precision Instruments
Insta-Weld Super Glue activator	Polyvance
Vetbond	3M, No.1469SB
Amoxicillin	Zoetis
0.9% Sodium Chloride	Pfizer 00409–4888
Dexamethasone	VetOne
RIMADYL (carprofen)	Zoetis
Lindocaine	VetOne
Cortex Buffer	7.888g NaCl, 0.372g KCl, 1.192g HEPES, 0.264g CaCL_2_, 0.204g MgCL_2_ in 1000ml millipore water

## Surgical procedure

**Fig 4:**
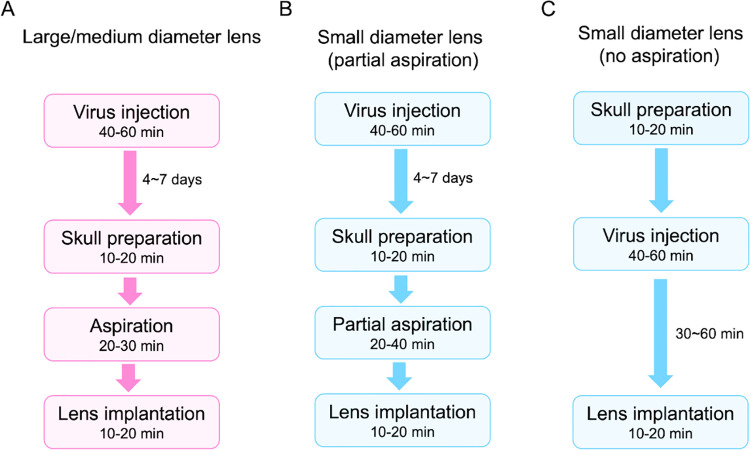
Diagram of different surgical procedures. (A) Procedure for the implantation of large/medium size lenses. (B) Procedure for the implantation of small diameter lens with partial aspiration. (C) Procedure for the implantation of small diameter lens without aspiration.

Depending on the brain region and the size of the lens implanted, there are three different implantation procedures that one can follow (Fig 4). To implant a lens with large or medium diameters (1.8 mm or 1 mm), aspiration is necessary to remove brain tissue. In order not to aspirate injected virus, we suggest that lens implantation takes place 4–7 days after viral injection (Fig 4A). To implant lenses with a small diameter (0.5 mm or 0.6 mm) (Fig 4C), aspiration can be skipped. Partial aspiration (Fig 4B) during thin lens implantation surgeries may be helpful to decrease imaging background, because one photon microscope has limited penetration depth, half aspiration lowers the chance to have tissue stacked under the lens compared to no aspiration procedure. Virus injection takes 40–60 minutes. Single lens implantation takes 1–1.5 hours (not including virus injection). Multiple lens implantation takes 1.5–2 hours.

1 **1.8 mm or 1 mm diameter lens implantation** (S1 and S2 Figs, step-by-step protocol in S1 File)

After injecting the virus, close up the skin with VetBond. Wait for 4–7 days, bring back the mouse, clean the surgical area and remove a 0.75 cm diameter circle of skin to expose the skull. Score the skull with a scalpel and disconnect muscles around neck to reduce the pull force on the skull and prevent muscle growth. Screw a surgical screw into a drilled hole in the contralateral hemisphere to the one where the lens would be implanted. Align bregma and lambda height and left/right hemisphere height. Locate and mark x, y coordinates of where the center of the lens will be placed (shift 100–200um from injection site). Measure a radius length of lens diameter (0.9 for 1.8 mm lens, 0.5 mm for 1 mm lens) medial/lateral/caudal/rostral of the labelled center and mark four dots. Drill an outline of the lens hole and remove this piece of skull (S1 and S2 Figs).

For implanting 1.8 mm lens into dCA1, start aspiration with a 27G blunt needle while continuously flushing with cortex buffer. Remove tissue until reaching corpus callosum marked by horizontal striations. Switch to 30G blunt needle and slowly peel these horizontal fibers and a few of diagonal fibers until vertical striations are visualized (S1 Fig). For implanting 1 mm lens into mPFC, start with a 27G blunt needle to aspirate the tissue with a thickness of 1 mm and switch to 30G blunt needle (S2 Fig).

Keep flushing the cavity with cortex buffer during aspiration. When bleeding stops, connect vacuum to homemade lens holder (for 1.8mm lens or 1mm lens) or insert lens into commercial lens holder (for 1 mm lens) (Fig 1). Move the lens to the top of cavity and lower it quickly to prevent bleeding. The lens should be implanted 200–300um above where virus was injected to consist with the working distance of the lens. Apply a small amount of cyanoacrylate glue around the bottom of the lens and the surrounding skull area. Remove lens holder carefully. Cover the exposed skull and seal the edges of the skin with cyanoacrylate glue. Apply dental cement to cover the whole area. Apply Kwik Sil on top of the lens to protect it (S1 and S2 Figs).

1 mm diameter and 4 mm long lens can also be implanted into dCA1 combined with V4 miniscope. All steps are the same as 1.8 mm lens implantation except changing the cavity diameter.

2 **Small diameter lens implantation with partial aspiration** (S3 Fig, step-by-step protocol in S1 File)

When implanting a thin lens (0.5 mm or 0.6 mm diameter) into deep brain regions, removing all the tissue is not practical because it is very difficult to clearly see the bottom of the cavity to make sure there is no bleeding or blood clots. Blindly, it is also difficult to make a cylinder shape aspiration. Therefore, we provide an alternative protocol for small diameter lens implantation with partial aspiration.

Start implanting lens 4–7 days after virus injection. Clean the surgical area, score the skull and fix a skull screw. Align bregma and lambda height and left/right hemisphere height. Locate and mark where the center of the lens will be placed (shift 100–200 μm from injection site if there is enough space). Mark four dots along future lens contour. Drill at the labelled lens center and extend to labelled outline dots. Infuse cortex buffer continuously to prevent overheating. Start aspiration with 27G blunt needle (mark 1 mm from the tip) until reach 1 mm deep. Switch to 30G blunt needle (mark about half of implantation depth from the tip) for more aspiration till mark on the needle reaches the level of cavity edge. Use a sharp needle to make a track for lens implant and retract it. Wash and clean the cavity using aspiration system with 30G needle until there is no more bleeding. Hold the lens with commercial holder and quickly lower the lens to the final position which is 200–300 μm above where virus was injected. Apply a small amount of cyanoacrylate glue around the bottom of the lens and attached skull area. Remove lens holder carefully and then cover the exposed skull and seal the edge of skin with cyanoacrylate glue. Apply dental cement to cover the whole surgical area. Apply Kwik Sil on top of the lens to protect it (S3 Fig).

3 **Small diameter lens implantation without aspiration** (step-by-step protocol in S1 File)

When implanting GRIN lenses with small diameters such as 0.5 mm and 0.6 mm, it is possible to entirely skip aspiration of the overlying tissue. In this case, virus injection and lens implantation can be combined into one surgery to help preventing off-target lens implantation and to make the surgical procedure shorter and easier.

Remove hair and clean the surgical area. Remove a round piece of skin (~0.75 cm diameter). Make scratches on the skull with a scalpel and use the scalpel to disconnect the muscles around neck in the surgical area. Screw a skull screw on the contralateral hemisphere skull. Align bregma and lambda height and left/right hemispheres. Locate and mark x and y coordinates of virus injection site and where the center of the lens will be placed (shift 100–200 μm from injection site if there is enough space). Mark four dots along future lens contour. Start drilling at the labelled lens center and extend to labelled outline dots. Infuse cortex buffer to prevent overheating. Inject virus into target area.

After virus injection, wait for 30–60 min, use a sharp needle to make a track for lens implant and retract it. Wash and clean the drilled hole using aspiration system with 30G needle until there is no more bleeding. Hold the lens with commercial holder and lower the lens quickly to the final position which is 200–300 μm above where virus was injected. Apply a small amount of cyanoacrylate glue around the bottom of the lens and attached skull area. Remove the lens holder carefully and then cover the exposed skull and seal the edge of skin with glue. Apply dental cement to cover the whole surgical area. Apply Kwik Sil on top of the lens to protect it.

4 **Two lenses implantation**

The UCLA Miniscope team has developed a large field of view miniaturized microscope, MiniXL, that allows imaging multiple GRIN lenses simultaneously [8]. Here, we provide methods of implanting two lenses in bilateral mPFCs or implanting one lens in mPFC and the other in NAc.

4.1 **Bilateral mPFC lens implantation** (S4 Fig, step-by-step protocol in S1 File)

Surgical procedures for bilateral mPFC lens implantations are similar to the large/medium diameter lens implantation methods described above, with some exceptions.

Bilaterally inject virus into mPFC (prelimbic PFC, PL) and wait for 4–7 days. To implant lens, remove hair and clean the surgical area. Remove a round piece of skin. Fix a skull screw on either hemisphere (visual cortex which is far from PFC is recommended). Align bregma and lambda heights and left/right hemispheres heights. Locate and mark where the centers of the lenses will be placed on both left and right hemispheres skull. Measure a radius length of lens diameter (0.5 mm for 1 mm lens) medial, lateral, caudal and rostral of the labelled centers and mark four dots in each hemisphere. For both hemispheres, drill the outline of the lens contour. Infuse cortex buffer to prevent overheating and to soften the thinned skull. Using fine forceps carefully lift and remove this piece of skull on each hemisphere. Briefly clean the skull and stop bleeding. As described for 1 mm lens implantation in mPFC, aspirate one cavity and implant lens into it. Apply a small amount of glue around the bottom of the lens and attached it to the skull. Be careful not to get glue into the contralateral cavity when applying glue. Repeat aspiration and implantation on the other hemisphere. Cover the whole exposed skull with cyanoacrylate glue and seal the edge of skin. Before glue completely dries, apply dental cement to cover the whole area and build four ‘walls’ surrounding two lenses. Apply Kwik Sil on top of both lenses to protect them (S4 Fig).

4.2 **Two-lens implantation into the mPFC and NAc** (S5 Fig, step-by-step protocol in S1 File)

Surgical procedures are mostly similar to the small diameter lens implantation without aspiration methods discussed above, with some exceptions. Here, we have selected 0.5 mm/6.1 mm lens for mPFC and 0.5 mm/8.4 mm lens for NAc.

Remove hair and clean the surgical area. Remove a round piece of skin. Make scratches on the skull and fix a skull screw. Align bregma and lambda height and left/right hemispheres. Locate and mark x and y coordinates of virus injection sites and where the centers of the lenses will be placed above mPFC and NAc. Drill two 0.5 mm diameter holes and perform viral injections into two sites. After virus injection, wait for 30–60 min. Insert a flattened needle to make a track in the cavity above NAc and retract it. Rinse the cavity with cortex buffer and Implant lens. Apply a small amount of cyanoacrylate glue and dental cement around the bottom of the lens upon skull to secure the lens. Repeat the same process for mPFC lens implant. Apply glue and dental cement on the skull and build four ‘walls’ surrounding two lenses. Apply Kwik Sil to cover both lenses (S5 Fig).

## Expected results

**Fig 5:**
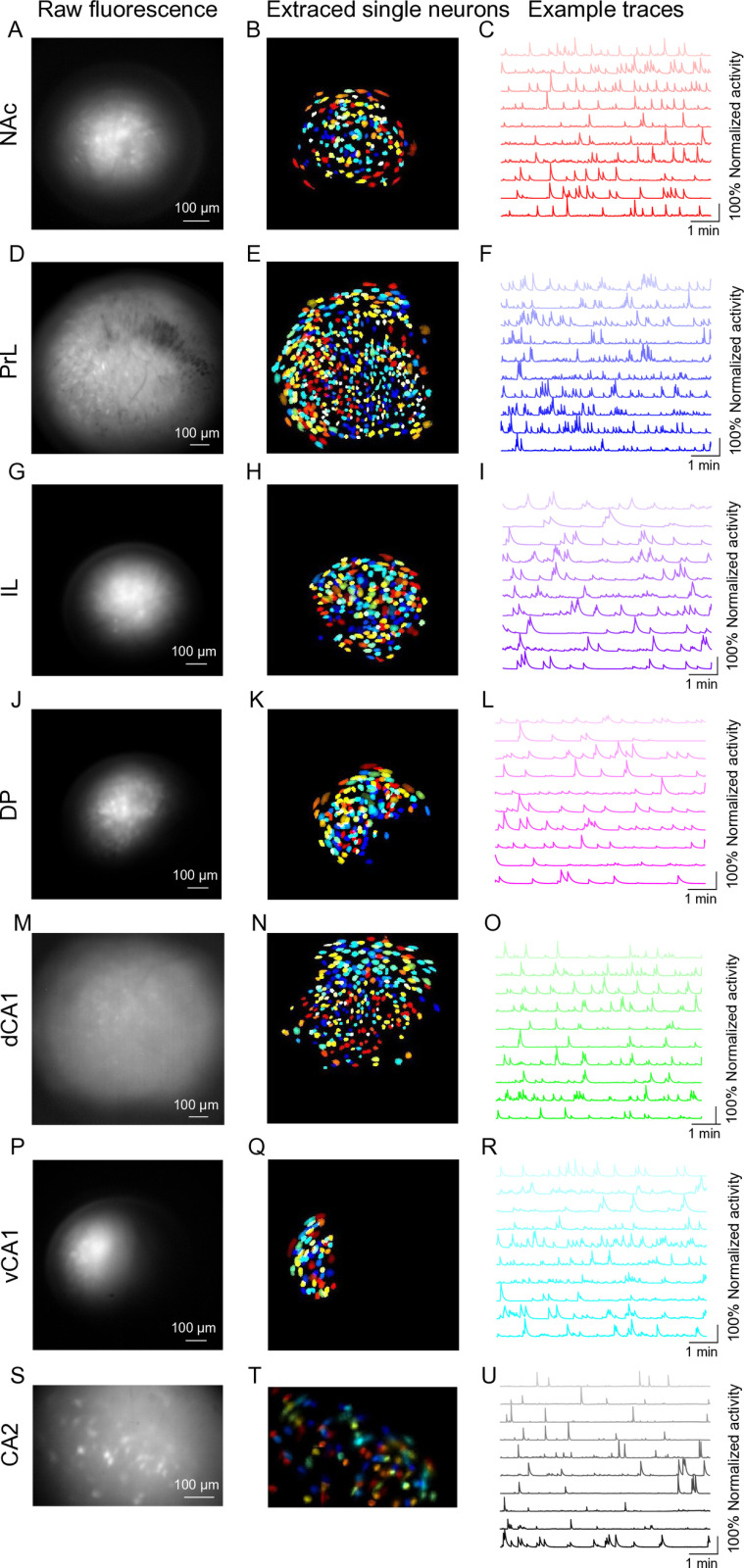
Raw imaging, extracted cells and activity traces. **(A–C)** Calcium imaging recorded in NAc through a 0.6 mm in diameter and 7.3 mm long relay lens by Miniscope V4, 154 neurons. **(D–F)** Calcium imaging recorded in PrL through a 1 mm in diameter and 4 mm long relay lens by Miniscope V4, 536 neurons. (G–I) Calcium imaging recorded in in IL through a 0.5 mm in diameter and 6.1 mm long relay lens by Miniscope V4, 193 neurons. **(J–L)** Calcium imaging recorded in DP through a 0.5 mm in diameter and 6.1 mm long relay lens by Miniscope V4, 151 neurons. **(M–O)** Calcium imaging recorded in dCA1 through a 1 mm in diameter and 4 mm long relay lens by Miniscope V4, 279 neurons. **(P–R)** Calcium imaging recorded in vCA1through a 0.5 mm in diameter and 6.1 mm long relay lens by Miniscope V4, 66 neurons. **(S–U)** Calcium imaging recorded in CA2 through a 1 mm in diameter and 4 mm long relay lens by Miniscope V3, 97 neurons. All Left panels, raw calcium fluorescence frames of the field of view (FOV) from example animals; All middle panels, single neurons extracted from the FOV; All right panels, calcium traces of ten example cells recorded from these brain regions. Mice were six to eight weeks old when surgeries were performed.

Here, we show example imaging from various brain regions including NAc, mPFC (PrL, IL), DP, dCA1, vCA1and CA2 using the UCLA Miniscope V4 or V3 (Fig 5). We have provided more details including calcium indicator, virus information, coordinates for virus injection and lens implantations, etc. in S1 Table. After lens implantation, mice are sent back to the vivarium for about three weeks recovery. Experimenters can then perform baseplating, habituation and recording. For Miniscope V4, parameters including LED intensity, recording frequency, gain value and focal plane are set in GUI (https://github.com/Aharoni-Lab/Miniscope-DAQ-QT-Software/wiki). For Miniscope V3, except manually adjusting focal plan, other parameters can be set in its GUI (https://github.com/daharoni/Miniscope_DAQ_Software). The NoRMCorre algorithm [22] is applied to perform motion correction. Constrained non-negative matrix factorization for microendoscopic data (CNMF-E) [23] is used to identify and extract the spatial shapes and fluorescent calcium activity of individual cells. We also suggest MiniAn [9] which is an analysis pipeline and visualization tool specifically for miniscope data. Overall, larger lenses and more superficial locations result in a larger number of neurons that can be recorded.

**Fig 6:**
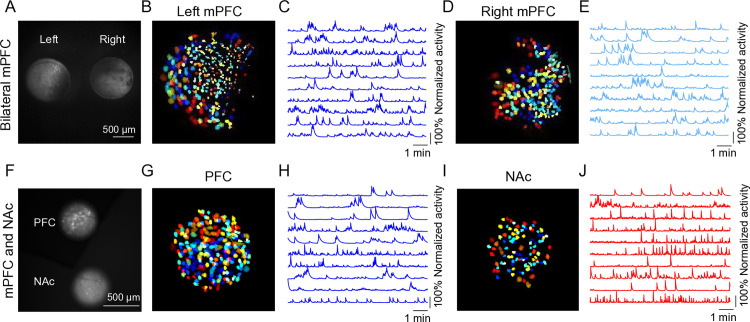
Multi-region imaging using MiniXL. **(A–E)** Bilateral mPFC calcium imaging through 1 mm in diameter and 4 mm long relay lenses by MiniXL. **(F–J)** PFC & NAc calcium imaging through 0.5 mm in diameter and 6.1 mm long relay lens (PFC) and 0.5 mm in diameter and 8.4 mm long relay lens by MiniXL. A and F, raw calcium fluorescence frames of the field of view (FOV) from example animals; B, D, G and I, single neurons extracted from the FOV; C, E, H and J, calcium traces of ten example cells. Mice were six to eight weeks old when surgeries were performed. More details can be found in S1 Table.

Taking advantage of our recently developed new large field of view Miniscope, MiniXL [8], we also show multi-brain region calcium imaging in freely behaving animals including bilateral mPFC imaging and PFC-NAc imaging (Fig 6). The same GUI as for UCLA Miniscope V4 is used for MiniXL recording. The same analysis packages (NoRMCorre and CNMF-E) are used for data analysis.

For multiple brain regions imaging, especially imaging different brain regions, it is critical to calculate the proper lens height such that the tops of the two lenses are precisely at the same level. Another issue that may arise is that there may be different viral expression levels at the two sites. Adjusting the virus dilution ratios may help. Also, Neutral Density (ND) Filters (Kodak) can be used to reduce the light emerging from one of the lenses (the brighter one) such that the same excitation strength can be used to record from both regions.

## Discussion

Over the past decade, different types of miniature microscopes have been developed with lighter weight [8,24,25], larger field-of-view [7,8,25], electronic focal plane adjustment [1,7,8] and flexible cabling for transferring power and data [1,7,8]. These tools have enabled studies of neural encoding of freely behaving during cognitive [4] and social tasks [5,14] as well as across different sleep/wake states [26,27]. We hope that this detailed and comprehensive protocol for different GRIN lens implantations in different brain regions will be valuable to neuroscience community.

To successfully record neural activity from interested brain regions during animal freely moving behavior, lens implantation is a key step, and choosing an appropriate strategy is crucial (S6 Fig). Since most of the GRIN lenses on the market have similar Numerical Aperture (NA, ~ 0.5), the cellular resolution would be decided by the miniscope model. With the same lens and device, imaging quality will depend on 1, lens size; 2, surgical procedure. Usually on the edge of the lens, cells will be distorted and may be excluded from dataset. Although we can record more cells through a lens with larger diameter, it is necessary to take the tissue lesion into consideration which may affect animal behavior. Thin lenses can help minimize lesions. Once decide which lens will be implanted, the experimenter must decide on how to implant it. Full aspirations are required for large/medium diameter lenses (1.8 mm, 1 mm) implanted in relative superficial subcortical areas. Both half aspiration and no aspiration are feasible for small diameter lenses (0.5 mm, 0.6 mm) implanted in deeper brain regions (DV > 2 mm). Since one photon microscopes have limited penetration depth and excite fluorophores throughout the entire light path, thus leading to higher background noise compared to two photon microscopes, half aspiration here can help decrease background noise. However, it can ease and shorten the procedure of lens implantation if we skip aspiration. These are trade-offs that the experimenters need to consider carefully. A pilot experiment is strongly recommended.

For those who are just beginning to attempt miniscope imaging, we suggest learning the surgery starting from dCA1 region. At dCA1, a relatively large cavity can help experimenters observe the condition of the cavity and there are clear landmarks which can help experimenters practice precise aspiration. During the surgical procedure, avoid touching any blood vessel. If there is excessive bleeding when aspiration is done, continuously flushing cortex buffer. Do not touch tissue when you aspirate the buffer. If the bleeding happens during implantation, remove the lens, clear the cavity using cortex buffer and try a second implantation. After surgery, post-operative care includes administering Carprofen and Dexamethasone. Amoxicillin is given in drinking water. We suggest monitoring animal health and behavior daily throughout the entire experimental period.

In this protocol, we also describe several innovative multiple lens implantation methods for multi-region imaging. The key point to get the experiment successful is accurately calculating the dorsal-ventral depths of multiple regions and making sure the tops of two or more lenses are within the same level. If only experimenters can use any tool to hold lenses vertically during implantation, they can get the tops of two or more lenses at the same height. In such a case, multiple areas should be able to be focused simultaneously. With our newly developed MiniXL, once the baseplate is properly secured on the skull, we can continuously track neuronal activity across many days with only slight adjustment of parameter setting refer to that of the previous session. According to the methods provided here, researchers can further test different combinations of brain regions and distinct genetic expression systems to assay cell-type and projection specific labeling.

Overall, we hope this protocol will increase the adoption of this technology and accelerate the use of novel surgical techniques.

## Supporting information

S1 File Step-by-step protocol, also available on protocol.io. (PDF)

S1 Fig Implant 1.8 mm diameter GRIN lens in dorsal hippocampal CA1 region. (A-D) Virus injection. (E-H) Skull preparation. (I-K) Aspiration procedure. (L-N) Lens implantation. (O, P) Protection of implanted lens.
(TIF)

S2 Fig Implant 1 mm diameter Relay lens in mPFC (prelimbic cortex). (A) Skull preparation. (B) Aspiration procedure. (C-F) Lens implantation. (G-I) Protection of implanted lens.
(TIF)

S3 Fig Implant 0.6 mm diameter relay lens in NAc. (A) Skull preparation. (B) Partial aspiration. (C) Insert a flattened needle to make a track for lens. (D,E) Lens implantation. (F-I) Protection of implanted lens.
(TIF)

S4 Fig Implant lenses in bilateral mPFCs. (A) Skull preparation. (B) Left mPFC aspiration. (C, D) mPFC lens implantation in left hemisphere. (E) Right mPFC aspiration. (F) mPFC lens implantation in right hemisphere. (G, H) Protection of implanted lenses.
(TIF)

S5 Fig Dual lens implantations in ipsilateral mPFC (PL) and NAc. (A) Skull preparation. (B-D) NAc lens implantation. (E and F) mPFC lens implantation. (G-H). Protection of implanted lenses.
(TIF)

S6 Fig Decision tree on GRIN lens implantation strategies. (TIF)

S1 Table Specific information about virus injection and lens implantation. (XLSX)

S1 Video Raw video of miniscope imaging in NAc (related to Fig 5A). (AVI)

S2 Video Raw video of miniscope imaging in PrL (related to Fig 5D). (AVI)

S3 Video Raw video of miniscope imaging in IL (related to Fig 5G). (AVI)

S4 Video Raw video of miniscope imaging in DP (related to Fig 5J). (AVI)

S5 Video Raw video of miniscope imaging in dCA1 (related to Fig 5M). (AVI)

S6 Video Raw video of miniscope imaging in vCA1 (related to Fig 5P). (AVI)

S7 Video Raw video of miniscope imaging in CA2 (related to Fig 5S). (AVI)

S8 Video Raw video of miniscope imaging in bilateral mPFCs (related to Fig 6A). (AVI)

S9 Video Raw video of miniscope imaging in NAc and mPFC (related to Fig 6F). (AVI)

## References

[1] Shuman T, Aharoni D, Cai DJ, Lee CR, Chavlis S, Page-Harley L, et al. Breakdown of spatial coding and interneuron synchronization in epileptic mice. Nat Neurosci. 2020;23(2):229–38. doi: 10.1038/s41593-019-0559-0 31907437 PMC7259114

[2] Gonzalez WG, Zhang H, Harutyunyan A, Lois C. Persistence of neuronal representations through time and damage in the hippocampus. Science. 2019;365(6455):821–5. doi: 10.1126/science.aav9199 31439798

[3] Ziv Y, Burns LD, Cocker ED, Hamel EO, Ghosh KK, Kitch LJ, et al. Long-term dynamics of CA1 hippocampal place codes. Nat Neurosci. 2013;16(3):264–6. doi: 10.1038/nn.3329 23396101 PMC3784308

[4] Cai DJ, Aharoni D, Shuman T, Shobe J, Biane J, Song W, et al. A shared neural ensemble links distinct contextual memories encoded close in time. Nature. 2016;534(7605):115–8. doi: 10.1038/nature17955 27251287 PMC5063500

[5] Zhao P, Chen X, Bellafard A, Murugesan A, Quan J, Aharoni D, et al. Accelerated social representational drift in the nucleus accumbens in a model of autism. bioRxiv. 2023:2023.08.05.552133. doi: 10.1101/2023.08.05.552133 37577515 PMC10418509

[6] Hur SW, Safaryan K, Yang L, Blair HT, Masmanidis SC, Mathews PJ, et al. Correlated signatures of social behavior in cerebellum and anterior cingulate cortex. eLife. 2024. doi: 10.7554/elife.88439.2 PMC10942583 38345922

[7] Guo C, Blair GJ, Sehgal M, Sangiuliano Jimka FN, Bellafard A, Silva AJ, et al. Miniscope-LFOV: A large-field-of-view, single-cell-resolution, miniature microscope for wired and wire-free imaging of neural dynamics in freely behaving animals. Sci Adv. 2023;9(16):eadg3918. doi: 10.1126/sciadv.adg3918 37083539 PMC10121160

[8] Zhao P, Guo C, Xie M, Chen L, Golshani P, Aharoni D. MiniXL: An open-source, large field-of-view epifluorescence miniature microscope for mice capable of single-cell resolution and multi-brain region imaging. bioRxiv. 2024:2024.08.16.608328. doi: 10.1101/2024.08.16.608328 39229051 PMC11370419

[9] Dong Z, Mau W, Feng Y, Pennington ZT, Chen L, Zaki Y, et al. Minian, an open-source miniscope analysis pipeline. Kemere C, Colgin LL, Kemere C, editors. Elife. 2022;11:e70661. doi: 10.7554/eLife.70661 35642786 PMC9205633

[10] Sehgal M, Filho DA, Kastellakis G, Kim S, Lee J, Martin S, et al. Co-allocation to overlapping dendritic branches in the retrosplenial cortex integrates memories across time. bioRxiv. 2021;2021.10.28.466343. doi: 10.1101/2021.10.28.466343

[11] Hattori R, Komiyama T. Longitudinal two-photon calcium imaging with ultra-large cranial window for head-fixed mice. STAR Protoc. 2022;3(2):101343. doi: 10.1016/j.xpro.2022.101343 35496806 PMC9048142

[12] Stetter C, Hirschberg M, Nieswandt B, Ernestus R-I, Heckmann M, Sirén A-L. An experimental protocol for in vivo imaging of neuronal structural plasticity with 2-photon microscopy in mice. Exp Transl Stroke Med. 2013;5(1):9. doi: 10.1186/2040-7378-5-9 23842538 PMC3716956

[13] Murugan M, Jang HJ, Park M, Miller EM, Cox J, Taliaferro JP, et al. Combined Social and Spatial Coding in a Descending Projection from the Prefrontal Cortex. Cell. 2017;171(7):1663-1677.e16. doi: 10.1016/j.cell.2017.11.002 29224779 PMC5889923

[14] Kingsbury L, Huang S, Wang J, Gu K, Golshani P, Wu YE, et al. Correlated Neural Activity and Encoding of Behavior across Brains of Socially Interacting Animals. Cell. 2019;178(2):429-446.e16. doi: 10.1016/j.cell.2019.05.022 31230711 PMC6625832

[15] Haggerty DL, Grecco GG, Reeves KC, Atwood B. Adeno-Associated Viral Vectors in Neuroscience Research. Mol Ther Methods Clin Dev. 2019;17:69–82. doi: 10.1016/j.omtm.2019.11.012 31890742 PMC6931098

[16] Xu X, Holmes TC, Luo M-H, Beier KT, Horwitz GD, Zhao F, et al. Viral Vectors for Neural Circuit Mapping and Recent Advances in Trans-synaptic Anterograde Tracers. Neuron. 2020;107(6):1029–47. doi: 10.1016/j.neuron.2020.07.010 32755550 PMC7530073

[17] Zhang Y, Rózsa M, Liang Y, Bushey D, Wei Z, Zheng J, et al. Fast and sensitive GCaMP calcium indicators for imaging neural populations. Nature. 2023;615(7954):884–91. doi: 10.1038/s41586-023-05828-9 36922596 PMC10060165

[18] Zhang Y, Looger LL. Fast and sensitive GCaMP calcium indicators for neuronal imaging. J Physiol. 2024;602(8):1595–604. doi: 10.1113/JP283832 36811153

[19] Masala N, Mittag M, Ambrad Giovannetti E, O’Neil DA, Distler F, Rupprecht P, et al. Aberrant hippocampal Ca2+ micro-waves following synapsin-dependent adeno-associated viral expression of Ca2+ indicators. 2024. doi: 10.7554/elife.93804.2 PMC11265795 39042440

[20] Daigle TL, Madisen L, Hage TA, Valley MT, Knoblich U, Larsen RS, et al. A Suite of Transgenic Driver and Reporter Mouse Lines with Enhanced Brain-Cell-Type Targeting and Functionality. Cell. 2018;174(2):465-480.e22. doi: 10.1016/j.cell.2018.06.035 30007418 PMC6086366

[21] Dana H, Chen T-W, Hu A, Shields BC, Guo C, Looger LL, et al. Thy1-GCaMP6 transgenic mice for neuronal population imaging in vivo. PLoS One. 2014;9(9):e108697. doi: 10.1371/journal.pone.0108697 25250714 PMC4177405

[22] Pnevmatikakis EA, Giovannucci A. NoRMCorre: An online algorithm for piecewise rigid motion correction of calcium imaging data. J Neurosci Methods. 2017;291:83–94. doi: 10.1016/j.jneumeth.2017.07.031 28782629

[23] Zhou P, Resendez SL, Rodriguez-Romaguera J, Jimenez JC, Neufeld SQ, Giovannucci A, et al. Efficient and accurate extraction of in vivo calcium signals from microendoscopic video data. Elife. 2018;7:e28728. doi: 10.7554/eLife.28728 29469809 PMC5871355

[24] Xue F, Li F, Zhang K, Ding L, Wang Y, Zhao X, et al. Multi-region calcium imaging in freely behaving mice with ultra-compact head-mounted fluorescence microscopes. Natl Sci Rev. 2024;11:nwad294. doi: 10.1093/nsr/nwad294 PMC10824555 38288367

[25] Scherrer JR, Lynch GF, Zhang JJ, Fee MS. An optical design enabling lightweight and large field-of-view head-mounted microscopes. Nat Methods. 2023;20(4):546–9. doi: 10.1038/s41592-023-01806-1 36928075

[26] Ingiosi AM, Hayworth CR, Harvey DO, Singletary KG, Rempe MJ, Wisor JP, et al. A Role for Astroglial Calcium in Mammalian Sleep and Sleep Regulation. Curr Biol. 2020;30(22):4373-4383.e7. doi: 10.1016/j.cub.2020.08.052 32976809 PMC7919541

[27] Blanco-Centurion C, Luo S, Spergel DJ, Vidal-Ortiz A, Oprisan SA, Van den Pol AN, et al. Dynamic Network Activation of Hypothalamic MCH Neurons in REM Sleep and Exploratory Behavior. J Neurosci. 2019;39(25):4986–98. doi: 10.1523/JNEUROSCI.0305-19.2019 31036764 PMC6670248

