# Supplementary material for: GRIN lens implantation strategies for in vivo calcium imaging using miniature microscopy
Source: PLoS One. 2025 May 12;20(5):e0323256. doi: 10.1371/journal.pone.0323256 (PMC12068630; doi:10.1371/journal.pone.0323256)
Supplement: S1 File — (PDF) [file pone.0323256.s001.pdf]

Mar 15, 2025

Protocol of GRIN lens implantation for in vivo calcium imaging using miniature microscopy

DOI
dx.doi.org/10.17504/protocols.io.ewov12jyogr2/v1

Pingping Zhao[1], Daniel Aharoni[1], Peyman Golshani[1]

[1]Department of Neurology, David Geffen School of Medicine, University of California, Los Angeles

Miniscope imaging

Pingping Zhao
UCLA

OPEN ACCESS

DOI: dx.doi.org/10.17504/protocols.io.ewov12jyogr2/v1

Protocol Citation: Pingping Zhao, Daniel Aharoni, Peyman Golshani 2025. Protocol of GRIN lens implantation for in vivo calcium imaging using miniature microscopy. **protocols.io https://dx.doi.org/10.17504/protocols.io.ewov12jyogr2/v1**

License: This is an open access protocol distributed under the terms of the **Creative Commons Attribution License**, which permits unrestricted use, distribution, and reproduction in any medium, provided the original author and source are credited

Protocol status: Working
We use this protocol and it's working

Created: March 14, 2025

Last Modified: March 15, 2025

Protocol Integer ID: 124445

Keywords: GRIN lens implantation, In vivo calcium imaging, Miniature microscopy, Brain, Medial prefrontal cortex, Hippocampus

Funders Acknowledgements:
NINDS
Grant ID: U01NS126050
NINDS
Grant ID: U01 NS122124
NIMH
Grant ID: 1R01MH132736-A1

Abstract

Miniature microscopy (Miniscope) has become one of the most popular and valuable neuroscience tools in the last decade. Miniscope in vivo calcium imaging during freely moving behavior has led to a number of transformative discoveries about neural coding across a large range of behaviors. The UCLA Miniscope Project is an open-source miniaturized microscopy platform that has greatly benefited the neuroscience community and led to the release of a number of different miniaturized microscopes with extended capabilities. While researchers can record from essentially any brain region through a cranial window or gradient Index of refraction (GRIN) lens, there is still a need for comprehensive protocols which describe detailed surgical procedures for successful miniaturized microscopy applications across different brain regions. Here, we provide step-by-step surgical procedures for implantation of GRIN lenses to record from a number of different brain regions including subregions in medial prefrontal cortex (PrL, IL, DP), subregions in hippocampus (dCA1, CA2 and vCA1), and ventral striatum (nucleus accumbens, NAc). Moreover, we also provide surgical methods of new multibrain regions imaging techniques developed by our group to record bilateral medial prefrontal cortex (mPFCs) or simultaneously record mPFC and NAc. Taken together, this protocol details easy and reproducible techniques for GRIN lens implantation and miniaturized microscopy in multiple structures.

Materials

Calcium indicator, virus information and surgical coordinates:

Brain region	Mouse strain	Calcium indicator	Virus information	Coordinates: Viurs injection	Coordinates: Lens implantation (Lens center)	Virus volume	Injection rate
NAc	A2A-Cre	GCaMP6f	pAAV.Syn.Flex.GCaMP6f.WPRE.SV40; Addgene 100833-AAV1	AP +1.4 mm, ML 0.87 mm, DV -4.5 mm	AP +1.4 mm, ML 0.75mm, DV -4.25 mm	400 nl	1 nl/s
PrL	CNTNAP2 KO	GCaMP6f	pAAV.Syn.GCaMP6f.WPRE.SV40; Addgene 100837-AAV1	AP +1.8 mm, ML 0.4 mm, DV -2.1 mm	Ap +1.8 mm, ML 0.5 mm, DV -1.8 mm	400 nl	
IL	VGlut1-Cre	GCaMP6f	pAAV.Syn.Flex.GCaMP6f.WPRE.SV40; Addgene 100833-AAV1	AP +1.7 mm, ML 0.4 mm, DV -2.85 mm	AP +1.7 mm, ML 0.35 mm, DV -2.65 mm	350 nl	
DP	VGlut2-Cre	GCaMP6f	pAAV.Syn.Flex.GCaMP6f.WPRE.SV40; Addgene 100833-AAV1	AP +2.1 mm, ML 0.6 mm, DV -3.4 mm	AP +2.1 mm, ML 0.6 mm, DV -3.2 mm	350 nl	
dCA1	C57	GCaMP6f	pAAV.Syn.GCaMP6f.WPRE.SV40; Addgene 100837-AAV1	AP -2.1 mm, ML 2 mm, DV -1.65 mm	AP -2.1 mm, ML 1.8 mm, DV -1.25 mm	500 nl	
vCA1	C57	GCaMP6f	pAAV.Syn.GCaMP6f.WPRE.SV40; Addgene 100837-AAV1	AP -3.16 mm, ML 3.5 mm, DV -3.7 mm	AP -3.16 mm, ML 3.5 mm, DV -3.5 mm	400 nl	
CA2	C57	GCaMP6f	PVN: pENN.AAV.hSyn.Cre.WPRE.hGH; Addgene 105553-AAV1.<br>CA2: pAAV.Syn.Flex.GCaMP6f.WPRE.SV40; Addgene 100833-AAV1	PVN: AP -0.94 mm, ML 0.22 mm, DV -4.9 mm<br>CA2: AP -1.6 mm, ML 1.6 mm, DV -1.7mm	CA2: AP -1.6 mm, ML 1.6 mm, DV -1.5mm	PVN: 150 nl<br>CA2: 300 nl	
Lfet & Right PrL	C57	GCaMP6f	Left & Right:<br>pAAV.Syn.GCaMP6f.WPRE.SV40; Addgene 100837-AAV1	Left: AP +1.8 mm, ML -0.4 mm, DV -2.1 mm<br>Right: AP +1.8 mm, ML 0.4 mm, DV -2.1 mm	Left: AP +1.8 mm, ML -0.5 mm, DV -1.8 mm<br>Right: AP +1.8 mm, ML 0.5 mm, DV -1.9mm	Left: 150 nl<br>Right: 300 nl	
PFC & NAc	C57	GCaMP6f	pAAV.Syn.GCaMP6f.WPRE.SV40; Addgene 100837-AAV1	PFC: AP +2mm, ML +0.5mm, DV -2.2mm<br>NAc: AP +1.3mm, ML +0.87mm, DV -4.5mm	PFC: AP +2mm, ML +0.3mm, DV -2mm.<br>NAc: AP +1.3mm, ML 0.9mm, DV -4.3mm	PFC: 400 nl<br>NAc: 400 nl	

GRIN lens:

- 1.8mm Dia, 1550nm DWL, 0.0mm WD, Uncoated, GRIN Lens **Edmund Optics worldwide Catalog #**64-531
- For PFC imaging, Inscopix 1050-004595, 4mm length and 1 mm diameter
- For NAc imaging, Inscopix 1050-004597, 7.3mm length and 0.6 mm diameter
- For bilateral PFC imaging, Inscopix 1050-004595, 4mm length and 1 mm diameter
- For multi-region imaging (mPFC and NAc), Inscopix 1050-004599, 6.1 mm length and 0.5 mm diameter (mPFC) and Inscopix 1050-004600, 8.4 mm length and 0.5 mm diameter (NAc)

UCLA Miniscope:

Miniscope V4 is required for imaging through relay lens (Inscopix 1050-004595, Inscopix 1050-004597, Inscopix 1050-004599, Inscopix 1050-004600). Miniscope V3 is required for imaging through 1.8mm GRIN lens (Edmund #64-531). New developed miniaturized microscope, MiniXL is required for multi-region calcium imaging through relay lens.

Lens holder:

Lens size (Diameter)	Holder	Information
1 mm / 1.8 mm	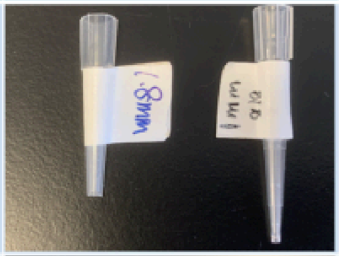	Homemade
1 mm	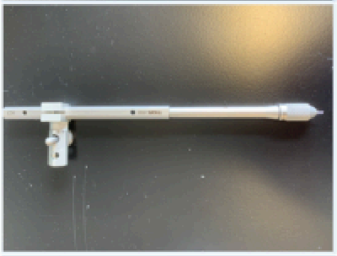	Thorlabs XCL
0.5 mm / 0.6mm	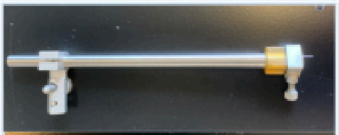<br>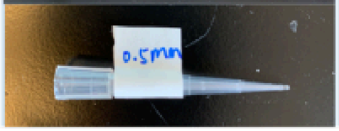	KORF Model 1770<br>Homemade

Aspiration system

Vacuum system includes tubes, a flask acting as a liquid trap and a syringe with a hole in its body to control vacuum.

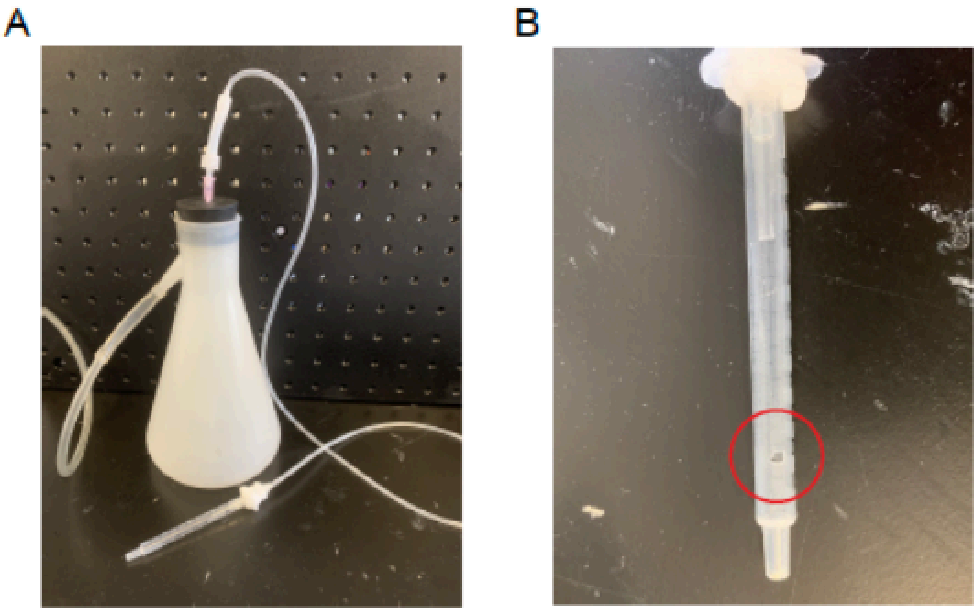

(A) Aspiration system is composed of a flask, the wall vacuum port, venous infusion needle and a 1 ml syringe. (B) Drill a hole on the side of syringe for controlling vacuum by finger pressing.

Surgical instruments information

Surgical instrument item	Information
Small animal stereotaxic instruments	KOPF Model 963
Mouse gas anesthesia head holder	KOPF 923B
Zygoma ear cups	KOPF 921
Surgical microscope	ZEISS Stemi 508
Isoflurane setup	SurgiVet
Homeothermic monitoring system	Harvard Apparatus
light	Zeiss
drill	MK-dent, HE17 Eco Line
Virus injection instrument	Drummond, NANOJECT III
FST tools	FST 14090-09; FST 10003-12; FSST 11223-20; FST 11295-10

Other supplies and reagents:

Item	Information
00-96 x 1/16 screw	Plastics One
27G needle	CMLsupply
30G needle	CMLsupply
31G needle	BD Veo U-100 syringes
Needle holder	KORF Model 1766
Marker pen	Sakura Pigma Micron pen 005
Cyanoacrylate glue	Krazy glue
Silicone Elastomer	Kwik-Sil (KWIK-SIL), World Precision Instruments
Insta-Weld Super Glue activator	Polyvance
Vetbond	3M, No.1469SB
Amoxicillin	Zoetis #055-085
0.9% Sodium Chloride	Pfizer 00409-4888
Dexamethasone	VET ONE 501012
RIMADYL (carprofen)	Zoetis #141-199
Lindocaine	VET ONE 510212
Cortex Buffer	7.888g NaCl, 0.372g KCl, 1.192g HEPES, 0.264g $CaCL_2$, 0.204g $MgCL_2$ in 1000ml milipore water

Animals:

Animal background, age, sex should be decided according to the experimental aim, but the current miniaturized microscopes are appropriate for adult (> 6 weeks old) mice. All mice were maintained on a 12h:12h light/dark cycle with food and water ad libitum. Mice were single housed for three to four weeks before in vivo calcium imaging and behavior experiments.

For surgeries involved in this study, mice were anaesthetized with 3-5% isofluraneoxygen mixture and placed into a stereotactic frame (David Kopf Instruments). Then 1- 2% isoflurane-oxygen mixture will be used throughout the surgery. Dexamethasone (0.2 mg/kg) and lidocaine (2%) will be administered 30 minutes before the start of surgery. During surgery, 1 ml 0.9% saline will be injected to prevent dehydration. The ophthalmic ointment will remain on the eyes until the animal is awake and ambulatory. After surgery, mice will be placed on a heating pad to maintain core temperature until it recovers from anesthesia. Post-operative care will involve administering Carprofen (5 mg/kg) injected subcutaneously, with a frequency of every 12-24 hours for 48 hours along with Dexamethasone (0.2 mg/kg). Additional doses may be administered after the initial 48- hour period as needed for pain. Amoxicillin (0.25 ml/mL) will be given in drinking water for 7 days. After the 7-day period, it will be replaced with normal water.

Part 1 Single Lens implantation: dCA1 (1.8 mm) and mPFC (1 mm) lens implantation 14m

1 Following institutional guidelines and lab protocols, anesthetize the animal and stabilize the head in the stereotaxic apparatus. Use forceps to open the mouse's mouth and put the upper incisors into the hole on palate bar. Slide the gas mask into position over the nose and tight the screw to hold the head stably.

Note

We use zygoma ear cups to further stabilize the head. Isoflurane and oxygen mixture (1.5-2%) is delivered through the metal mask. Use vacuum under the gas anesthesia head holder to prevent experimenters from exposure.

2 Remove hair at surgical area. Clean the surgical area by three alternating scrubs of ethanol and iodine. Using a pair of scissors to make an incision along midline. Remove fascia with cotton swab dipped in hydrogen peroxide (S1A and S1B Fig).

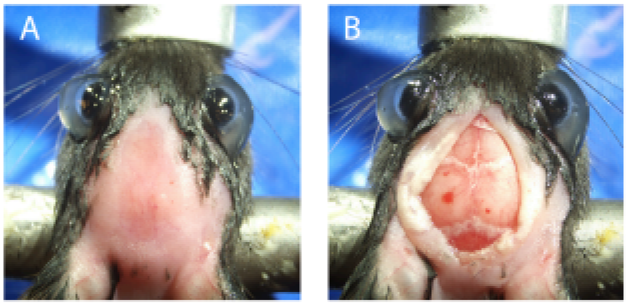

S1 Fig Implant 1.8mm diameter GRIN lens in dorsal hippocampal CA1 region. (A-D) Virus injection.

3 Level the head position by measuring z-position of bregma and lambda as well as left and right hemisphere (going left and right 1 mm from bregma to measure their heights). Adjust head position to equalize bregma and lambda position as well as leftright positions. Locate the injection site with x, y coordinates, label it by a FineLiner pen (MICRON) and drill a small hole at that position carefully. Cortex buffer can be applied to prevent overheating (S1C Fig).

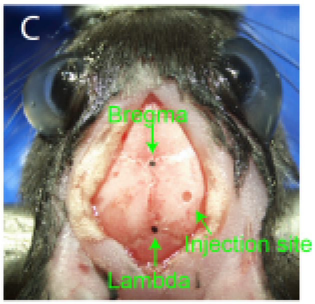

S1 Fig Implant 1.8mm diameter GRIN lens in dorsal hippocampal CA1 region. (A-D) Virus injection.

4 Using a Nanoject III device, inject virus at a speed of 1 nl/sec and hold the capillary pipette inside tissue for 00:10:00 . Typical injections are 100-500 nl. Retract the pipette and close up the skin with VetBond (3M) (S1D Fig). Inject Carprofen (5 µL) and 0.5 mL Saline afterwards. 10m

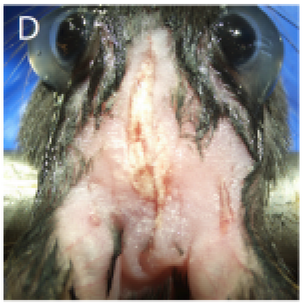

S1 Fig Implant 1.8mm diameter GRIN lens in dorsal hippocampal CA1 region. (A-D) Virus injection.

5 Monitor the mouse for recovery on heat pad before sending the mouse back to the vivarium.

6 Give 0.25 µL Amoxicillin in water to prevent infection and three days postoperation care with daily subcutaneous injections of carprofen (5 µL) for pain relief.

7 After 4-7 days, bring the mouse back to surgery room. Anesthetize the animal and stabilize the mouse head in the stereotaxic apparatus.

8 Remove hair at surgical area if there is hair regrown. Clean the surgical area with ethanol and iodine. Make incisions to remove a 0.75 cm diameter circle of skin (S1E Fig). Remove fascia with cotton swapped dipped in hydrogen peroxide.

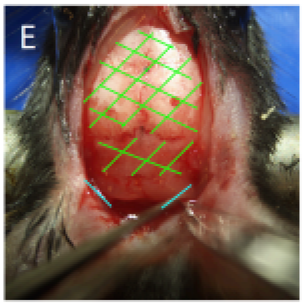

S1 Fig Implant 1.8mm diameter GRIN lens in dorsal hippocampal CA1 region. (E-H) Skull preparation.

9 Make multiple scratches on the skull with a scalpel and disconnect muscles around neck using scalpel to reduce pull on the skull and prevent muscle growth (S1E Fig). Use a Q-tip to dry and stop bleeding.

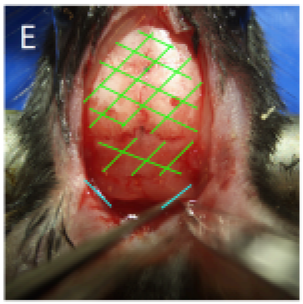

S1 Fig Implant 1.8mm diameter GRIN lens in dorsal hippocampal CA1 region. (E-H) Skull preparation.

10 Drill the skull in the hemisphere contralateral to the position of the lens (eg, contralateral visual cortex if implant lens in mPFC with 1 mm lens or dCA1 with 1.8 mm lens). Do not drill through the skull. Screw a surgical screw (shaft diameter, 1.6 mm) into the hole (S1F and S2A Fig). Stop screwing once surgical screw is stabilized in order not to disturb brain tissue underneath. Remember to check whether mouse head is still stabilized.

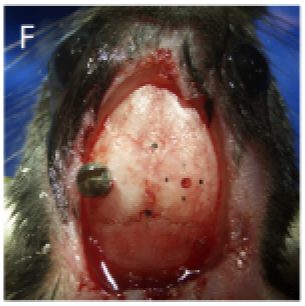

S1 Fig Implant 1.8mm diameter GRIN lens in dorsal hippocampal CA1 region. (E-H) Skull preparation.

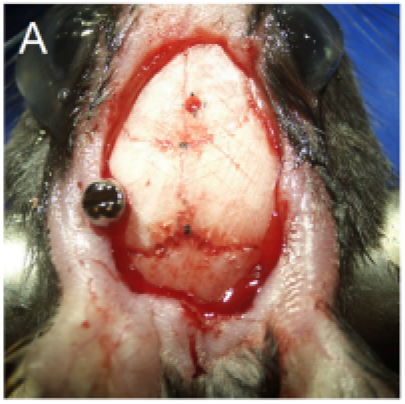

S2 Fig Implant 1mm diameter Relay lens in mPFC (prelimbic cortex). (A) Skull preparation.

11 Align bregma and lambda heights and left/right hemisphere heights as described in step 3. Locate and mark x, y coordinates of where the center of the lens will be placed. If the recording area allows, 100-200 µm shift of lens center from virus injection site is suggested. Measure a radius length of lens diameter (0.9 mm for 1.8 mm lens, 0.5mm for 1mm lens) medial/lateral/caudal/rostral of the labelled center and mark four dots (S1F and S2A Fig).

Note

During alignment, take note of the heights of the top of the skull at bregma and at where the center of the lens will be placed. Get the height difference between two sites. Because of the curve of skull and individual differences in animals, final implantation depth needs to be calibrated according to this difference for each mouse.

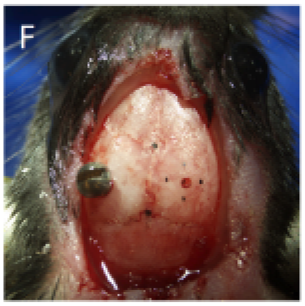

S1 Fig Implant 1.8mm diameter GRIN lens in dorsal hippocampal CA1 region. (E-H) Skull preparation.

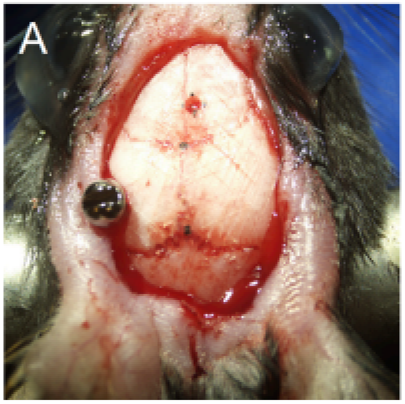

S2 Fig Implant 1mm diameter Relay lens in mPFC (prelimbic cortex). (A) Skull preparation.

12 To drill the outline of the lens hole, drill four marked dots first (S1G Fig) and extend to dots' both sides. Connect four arcs to a circle (S1H Fig). Do not drill through the skull and only thin the skull during this process. Infuse cortex buffer while drilling to prevent overheating and to soften the thinned skull. Gently touch and press this piece of skull to check whether it is just slightly connected to the surround part of skull. If it feels steady, further drilling is needed. Using a fine forceps to hold the edge of this round piece of skull and carefully lift and remove it (S1I Fig). Use the fine forceps to go around the edge of cavity to smooth it. Use a recycled lens to estimate whether the lens can into the drilled hole.

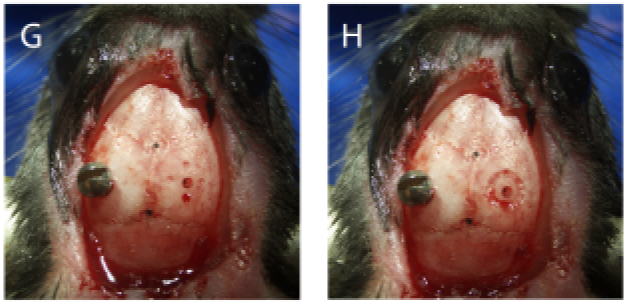

S1 Fig Implant 1.8mm diameter GRIN lens in dorsal hippocampal CA1 region. (E-H) Skull preparation.

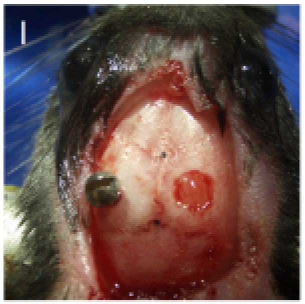

S1 Fig Implant 1.8mm diameter GRIN lens in dorsal hippocampal CA1 region. (I-K) Aspiration procedure.

13 Connect vacuum to aspirator. Start aspiration with a 27G blunt needle and continuously flushing with cortex buffer.

13.1 For implanting 1.8 mm lens into dCA1: Start aspiration with a 27G blunt needle to horizontally remove tissue until reaching corpus callosum marked by horizontal striations (S1J Fig). Continuously flushing with cortex buffer. Switch to 30G blunt needle and slowly peel these horizontal fibers and a few of the diagonal fibers. Stop aspiration when vertical striations are visualized and try to keep these striations (the alveus) intact (S1K Fig).

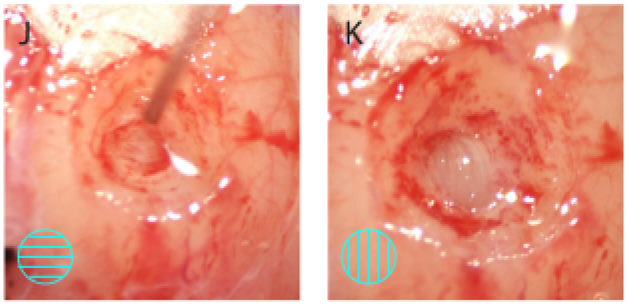

S1 Fig Implant 1.8mm diameter GRIN lens in dorsal hippocampal CA1 region. (I-K) Aspiration procedure.

13.2 For implanting 1 mm lens into mPFC (eg., PrL): Mark 27G blunt needle at 1 mm from the tip and mark the 30G blunt needle at 200-300 µm less than implantation depth from the tip. Start aspiration with a 27G needle to horizontally remove tissue until the marker reaches the skull edge of the cavity. Switch to 30G needle and slowly peel away more tissue until the marker reaches the skull edge of the cavity. There are no clear fiber tracks to guide aspiration in mPFC as there are in dCA1 (S2B Fig).

Note

During the whole aspiration process, try to make the cavity cylinder shaped instead of cone shaped and try to make the bottom aspiration as flat as possible. Remember to horizontally peel the tissue instead of vertically poking the tissue.

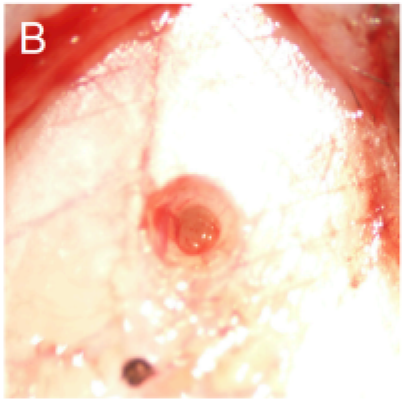

S2 Fig Implant 1mm diameter Relay lens in mPFC (prelimbic cortex). (B) Aspiration procedure.

14 Wait for 00:01:00 - 00:02:00 to make sure there is no more bleeding. If there bleeds again, keep flushing and sucking out cortex buffer without touching the tissue around. Wait for extra 00:01:00 - 00:02:00 , during this time, connect vacuum to the homemade lens holder (for 1.8 mm /1 mm lens) or insert lens into commercial lens holder (for 1 mm lens). Invert microscope and adjust it so that you can visualize the bottom of lens (S1L Fig). 4m

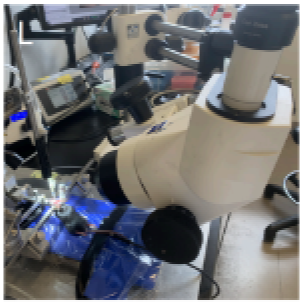

S1 Fig Implant 1.8mm diameter GRIN lens in dorsal hippocampal CA1 region. (L-N) Lens implantation.

15 Implant lens

15.1 For implanting 1.8 mm lens into dCA1: Move the held lens to the hole and lower it down where will be set as the local skull top (for 1.8 mm lens, medial half of the lens under the skull and lateral half of the lens upon the skull) (S1M Fig).

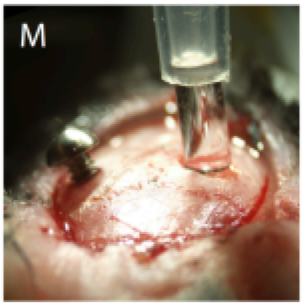

S1 Fig Implant 1.8mm diameter GRIN lens in dorsal hippocampal CA1 region. (L-N) Lens implantation.

15.2 For implanting 1 mm lens into the mPFC: Besides the method described above (step 15.1), another way to set zero of height for implantation is to move the held lens to bregma, gently touch bregma and record the height (S2C Fig). Then move the lens to the aspirated hole and

move it to the same height vertically (S2D Fig). With this method, no calibration is needed for implantation depth.

Note

Since 1 mm diameter is relatively big, it may not be precise to locate the center of the lens to bregma. Step 15.1 here will be strongly suggested for 1mm lens implantation (for 1 mm lens, make the lens bottom at the same level of the edge of cavity).

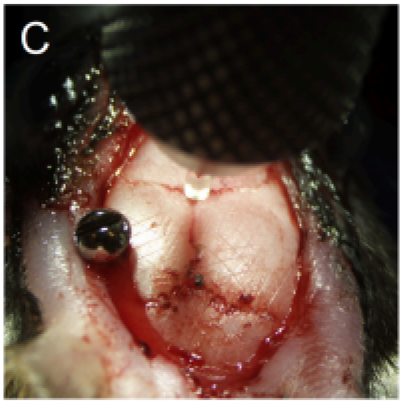

S2 Fig Implant 1mm diameter Relay lens in mPFC (prelimbic cortex). (C-F) Lens implantation.

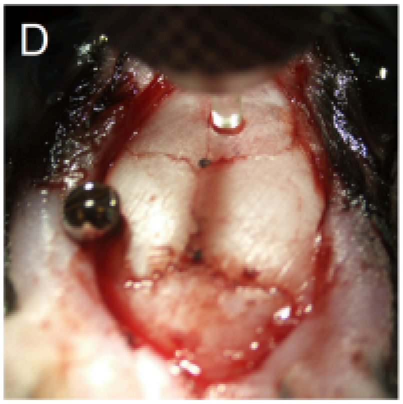

S2 Fig Implant 1mm diameter Relay lens in mPFC (prelimbic cortex). (C-F) Lens implantation.

Note

Lower the lens quickly to prevent bleeding (S1N, S2E Fig). If use method step 15.1 to set zero of z axis, the depth for inserting lens should be calibrated (as mentioned in step 11). The lens should be implanted 200-300 µm above where virus was injected consistent with the working distance of the lens.

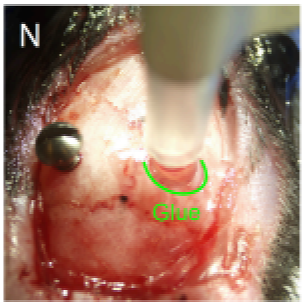

S1 Fig Implant 1.8mm diameter GRIN lens in dorsal hippocampal CA1 region. (L-N) Lens implantation.

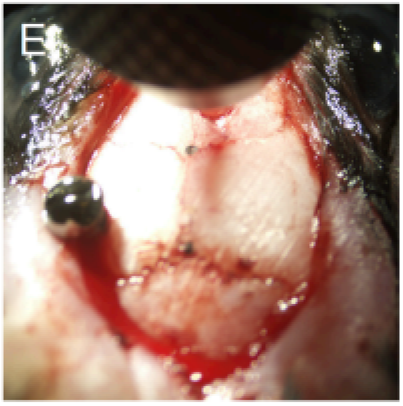

S2 Fig Implant 1mm diameter Relay lens in mPFC (prelimbic cortex). (C-F) Lens implantation.

16 Apply a small amount of cyanoacrylate glue around the bottum of the lens and attached skull area. Apply one drop of activator to dry the glue immediately (S1N, S2E Fig). Remove lens holder carefully (S1O, S2F Fig. For homemade lens holder, remove the vacuum tube first and then lift the holder.

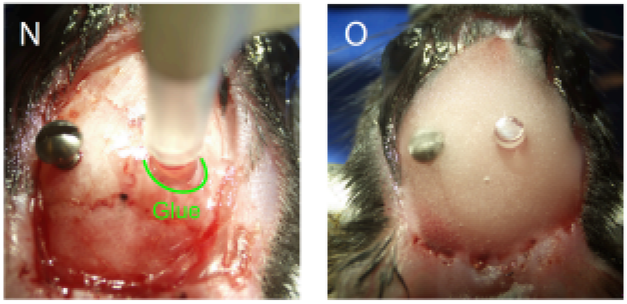

S1 Fig Implant 1.8mm diameter GRIN lens in dorsal hippocampal CA1 region. (L-N) Lens implantation. (O, P) Protection of implanted lens.

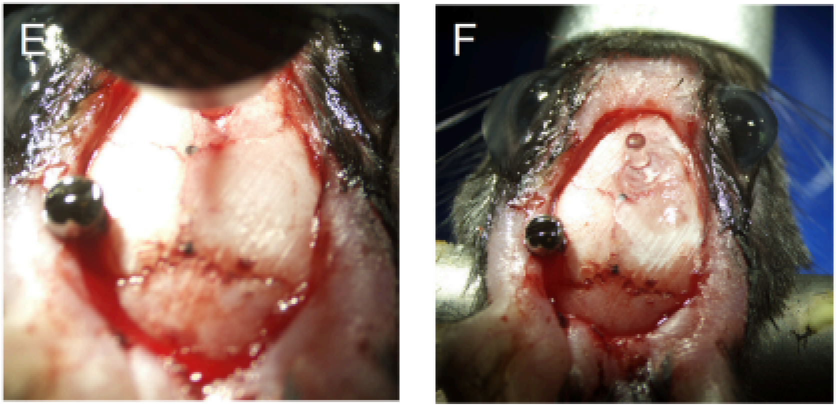

S2 Fig Implant 1mm diameter Relay lens in mPFC (prelimbic cortex). (C-F) Lens implantation.

17 Cover the exposed skull with cyanoacrylate glue and seal the edges of the skin. Before glue completely dries, apply dental cement to cover the whole area (S1O, S2G and S2H Fig). The thickness of dental cement should not be higher than the height of partial lens outside of the skull. We suggest applying thick dental cement to build four 'walls' around the lens (S1P, S2H Fig) which will facilitate baseplating.

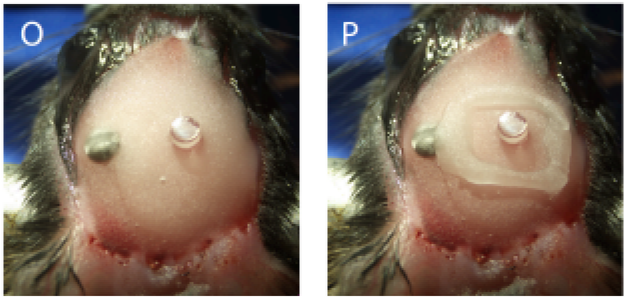

S1 Fig Implant 1.8mm diameter GRIN lens in dorsal hippocampal CA1 region. (O, P) Protection of implanted lens.

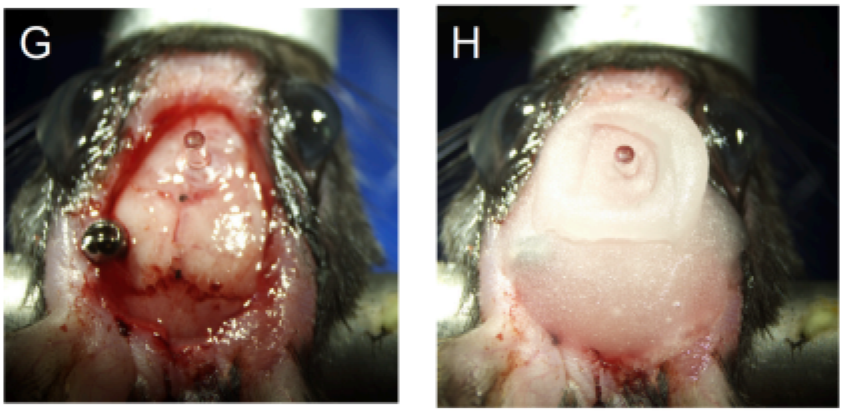

S2 Fig Implant 1mm diameter Relay lens in mPFC (prelimbic cortex). (G-I) Protection of implanted lens.

18 Apply Kwik Sil on top of the lens. Make sure it covers the whole lens. Four walls can also help stopping the flow of Kwik Sil (S2I Fig).

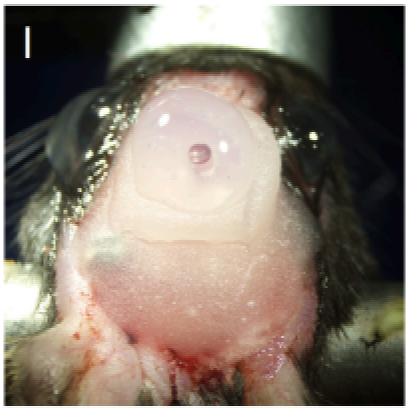

S2 Fig Implant 1mm diameter Relay lens in mPFC (prelimbic cortex). (G-I) Protection of implanted lens.

19 Inject 5 µL Carprofen, 0.2 µL Dexamethasone and 0.5 mL Saline subcutaneously and add 0.25 µL Amoxicillin into water.

20 Monitor the mouse for recovery on heating pad and transport it back to the vivarium.

21 Seven days post-operation care with subcutaneous injection 5 µL Carprofen and 0.2 µL Dexamethasone.

Note

1 mm diameter and 4 mm long lens can also be implanted into dCA1 combined with Miniscope V4. All steps are the same as 1.8 mm lens implantation except changing the cavity diameter.

Part 1 Single Lens implantation: Thin lens implantation in deep brain region with partial aspiration

2m

22 Inject virus in NAc as described in step 1 to step 10.

23 Align bregma and lambda heights and left/right hemisphere heights. Locate and mark x,y coordinates of where the center of the lens will be placed (shift 100-200 µm from injection site if there is enough space for the interested brain region). Measure a radius length of lens diameter (0.25 mm for 0.5 mm lens, 0.3 mm for 0.6 mm lens) medial/lateral/caudal/rostral of the labelled center and mark four dots. Calculate height difference between the skull tops of bregma and the labelled lens center.

24 Start drilling at the labelled lens center and extend to labelled outline dots. Flush cortex buffer to prevent overheating. Use a fine forceps to scratch around the edge of cavity to smooth it. Use a recycled lens to estimate whether the lens can fit into the cavity (S3A Fig).

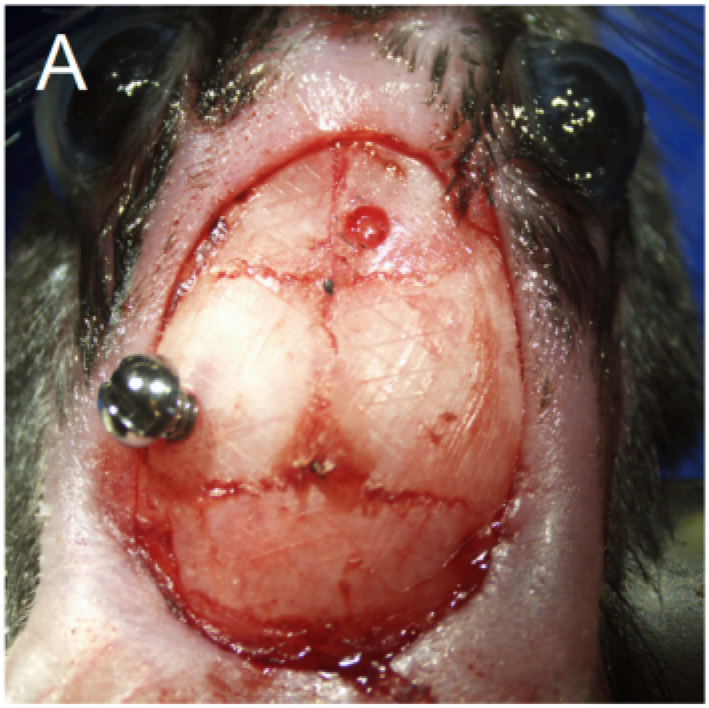

S3 Fig Implant 0.6 mm diameter relay lens in NAc. (A) Skull preparation.

25 Mark 27G blunt needle with a marker at 1 mm from the tip and mark 30G blunt needle with a marker at a length of about half of the depth of implantation. For example, for NAc implantation of 0.6 mm/7.3 mm lens, lens will be lowered to -4.3 mm. Mark 30G needle at ~2 mm from the tip (about 2 mm aspiration). Connect vacuum to aspirator (see Materials, Aspiration system). Start aspiration with a 27G blunt needle and continuously flushing with cortex buffer. When the marker reaches the edge of the skull, switch to 30G needle and carefully peel away more tissue until the marker reaches the edge of skull. Make sure there is no more bleeding and blood clot on the bottom (S3B Fig).

Note

During half aspiration process for thin lens implantation, try to make the cavity cylinder shaped instead of cone shaped. In order to check the bottom of the cavity, adjust the angle of surgical microscope to make it perpendicular to the horizontal plane.

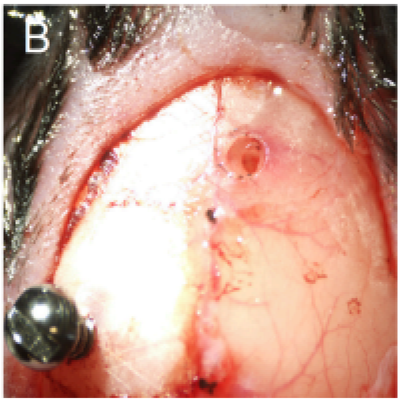

S3 Fig Implant 0.6 mm diameter relay lens in NAc. (B) Partial aspiration.

26 Move a flattened needle to bregma and measure the height. Move the needle to x,y coordinates of where lens center will be located (center of the cavity), lower it to the height measure at bregma skull top and set zero. Insert the needle into the hole and lower it to 200-300 µm above where the lens will be implanted (S3C Fig). For example, In NAc, 0.6 mm lens will be implanted at -4.3 mm (from bregma skull top). The needle will be lowered to -4 mm. Retract the needle. Clean the hole again using aspiration system with 30G needle. At this stage, only aspirate flushing cortex buffer. Do not touch the tissue on the bottom.

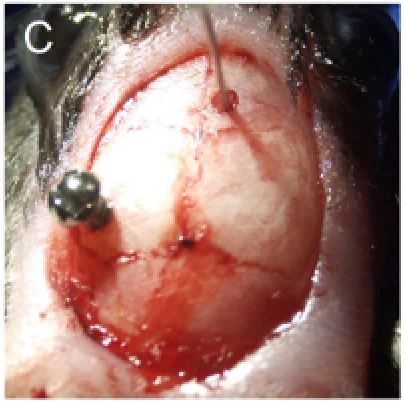

S3 Fig Implant 0.6 mm diameter relay lens in NAc. (C) Insert a flattened needle to make a track
for lens.

27 Wait for 00:01:00 - 00:02:00 to make sure there is no more bleeding. During this time, insert lens into commercial lens holder (see Materials, Lens holder), invert microscope and adjust it at an angle to monitor the bottom of the lens. 2m

28 Move the held lens to the top of the hole. Remember to adjust DV coordinate according to the height difference between the skull tops of bregma and labelled lens center at implantation site (S3D Fig). Lower the lens quickly to prevent bleeding. The lens should be implanted 200-300 µm above where virus was injected according to the working distance of the lens (S3E Fig). Step 15.2 can also be applied here.

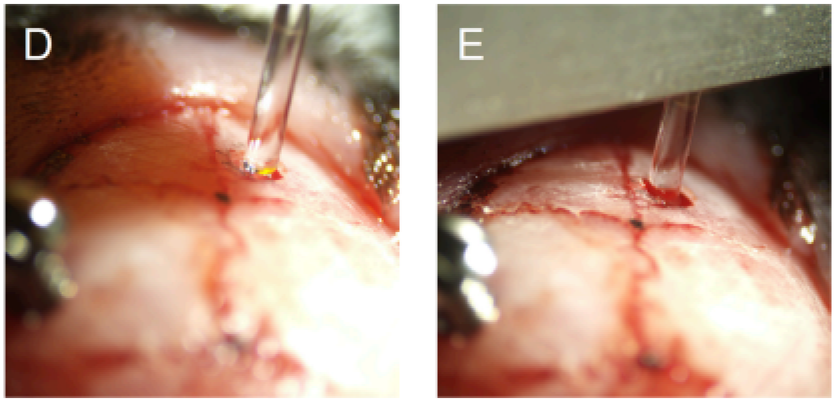

S3 Fig Implant 0.6 mm diameter relay lens in NAc. (D, E) Lens implantation.

29 Apply a small amount of cyanoacrylate glue around the bottom of the lens and attached skull area. Apply one drop of activator to dry the glue immediately. Remove lens holder carefully.

30 Cover the exposed skull with cyanoacrylate glue and seal the edge of skin (S3F Fig). Before glue completely dries, apply dental cement to cover the whole area (S3G Fig) and build four 'walls' as described in step 17 (S3H Fig).

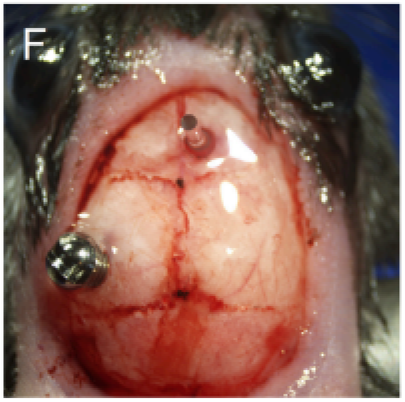

S3 Fig Implant 0.6 mm diameter relay lens in NAc. (F-I) Protection of implanted lens.

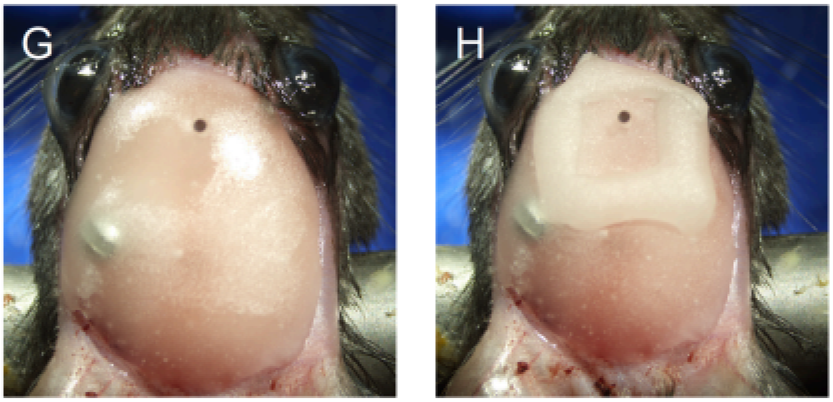

S3 Fig Implant 0.6 mm diameter relay lens in NAc. (F-I) Protection of implanted lens.

31 Apply Kwik Sil on top of the lens (S3I Fig).

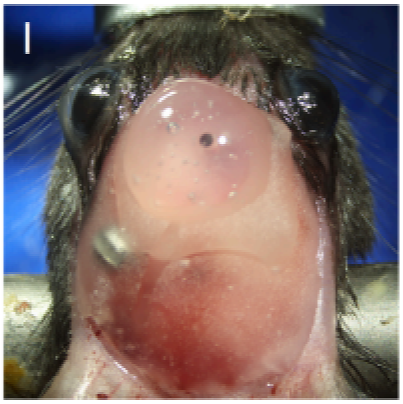

S3 Fig Implant 0.6 mm diameter relay lens in NAc. (F-I) Protection of implanted lens.

32 Inject 5 µL Carprofen, 0.2 µL Dexamethasone and 0.5 mL Saline subcutaneously. Add 0.25 µL amoxicillin into water.

33 Monitor the mouse for recovery on heat pad and transport to the vivarium.

34 Seven days post-operation care with subcutaneous injection 5 µL Carprofen and 0.2 µL Dexamethasone.

Part 1 Single Lens implantation: Thin lens implantation in deep brain region without aspiration

1h 10m

35 Following institutional guidelines and lab protocols, anesthetize the animal and stabilize the mouse head in the stereotaxic apparatus.

36 Remove all hair in the surgical area. Clean the surgical area by three alternating scrubs of ethanol and iodine. Make multiple incisions to remove a round piece of skin (~0.75 cm in diameter). Remove fascia with cotton swab soaked in hydrogen peroxide.

37 Score the skull with a scalpel and use the scalpel to disconnect the muscles around neck in the surgical area. Use Q-tips to dry and stop all bleeding.

38 Drill the contralateral skull and screw a surgical screw. Check whether mouse's head is still stabilized.

39 Align bregma and lambda heights and left/right hemisphere heights. Locate and mark x and y coordinates of virus injection site and where the center of the lens will be placed (shift 100-200 µm from injection site if there is enough space). Measure a radius length of lens diameter (0.25 mm for 0.5 mm lens, 0.3 mm for 0.6 mm lens) medial/lateral/caudal/rostral of the labelled center and mark four dots. Calculate height difference between the skull top of bregma and labelled future lens center.

40 Start drilling at the labelled lens center and extend to labelled outline dots. Flush some cortex buffer to prevent overheating. Use a recycled lens to estimate whether the GRIN lens can fit into the hole.

41 Insert the pipette to the correct coordinates. Inject virus at a speed of 1 nl/sec and hold the capillary pipette inside tissue for 00:10:00 . Retract the pipette and wait for 00:30:00 - 01:00:00 .

1h 10m

42 Use fine forceps to remove any blood clots and smooth the edge of the cavity. Flush the cavity with cortex buffer and suck it using 30G blunt needle connected to aspiration vacuum system.

43 Please repeat steps as described in step 26 to 34.

> **Note**
>
> Using a thin lens leads to less tissue damage and enables implantation without aspiration which makes the surgical procedure shorter and easier. However, overall, we have found that implantation without aspiration results in higher background.

Part 2 Two lenses implantation: Bilateral mPFC lens implantation

2m

44 Please repeat steps as described in step 1 to 10 (except perform bilateral instead of unilateral virus injection).

45 Align bregma and lambda heights and left/right hemisphere heights. Locate and mark x,y coordinates for where the center of the lenses will be placed on both left and right hemispheres of the skull. Measure a radius length of lens diameter (0.5mm for 1mm lens) medial, lateral, caudal and rostral of each labelled center and mark four dots in each hemisphere. Calculate height difference between the skull top of bregma and labelled lens centers.

46 For both hemispheres, drill the outline of lens holes. Flush cortex buffer to prevent overheating and to soften the thinned skull. Using fine forceps carefully lift and remove this piece of skull on each hemisphere. Go around the cavity edge and smooth it. Use a recycled lens to estimate whether each GRIN lens can fit into each hole. (S4A Fig).

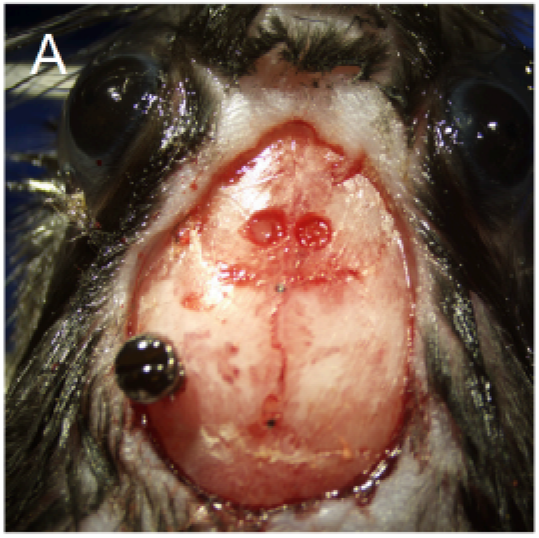

S4 Fig Implant lenses in bilateral mPFCs (A) Skull preparation

47 Mark a 27G blunt needle at 1mm from the tip. Mark a 30G blunt needle with a length of 200-300 μm less than implantation depth from the tip using a marker pen (e.g.: to implant lens at -1.8 mm, mark the needle at 1.5 mm). Connect vacuum to aspirator. Start aspiration with a 27G needle until the marker reaches the skull edge of the hole. Switch to 30G need and slowly peel away more tissue until the marker reaches the skull edge of the hole (S4B Fig).

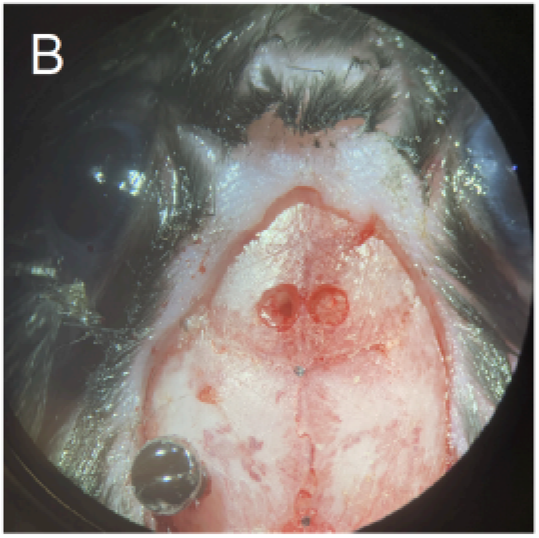

S4 Fig Implant lenses in bilateral mPFCs (B) Left mPFC aspiration.

48 Once bleeding has been stopped in one of the cavities, wait for 00:01:00 - 00:02:00 to make sure there is no more bleeding. Insert lens into lens holder (for 1 mm lens, see Materials, Lens holder), invert microscope and adjust the microscope head to an angle that allows experimenter to monitor the bottom of lens.

2m

Note

Because of the limited distance between two lenses, a homemade lens holder is recommended.

49 Move the held lens to the hole, lower it down to make the lens bottom at the same level of the edge of the hole and set z-position as zero. Lower the lens quickly to prevent bleeding (S4C Fig).

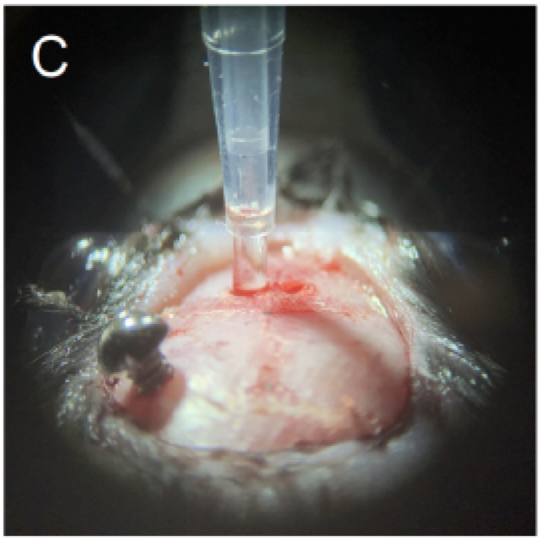

S4 Fig Implant lenses in bilateral mPFCs (C, D) mPFC lens implantation in the left hemisphere.

50 Apply a small amount of cyanoacrylate glue around the bottom of the lens. Apply one drop of activator to dry the glue immediately. Remove the lens holder carefully.

51 For contralateral implantation, repeat step 48-50 (S4E and S4F Fig).

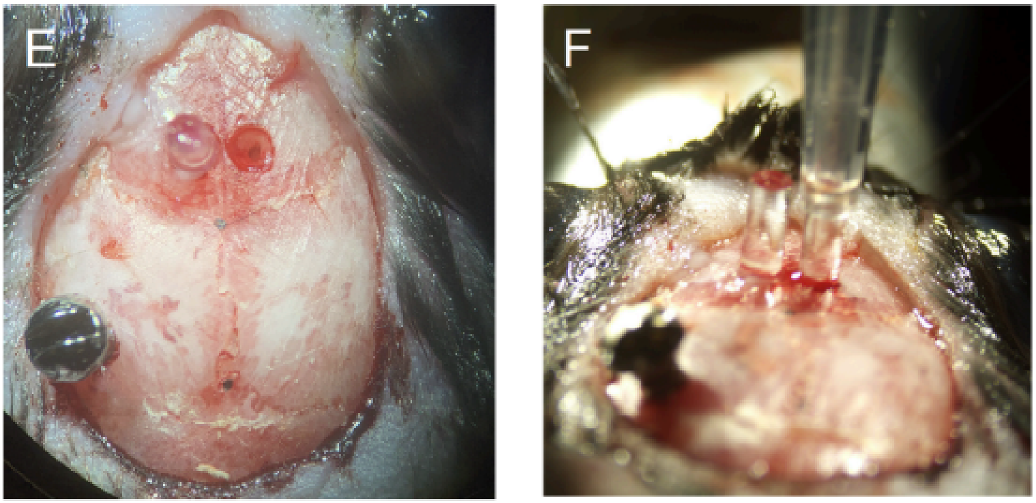

S4 Fig Implant lenses in bilateral mPFCs (E) Right mPFC aspiration. (F) mPFC lens implantation in the right hemisphere.

52 Cover the whole exposed skull with cyanoacrylate glue and seal the edge of skin (S4G Fig). Before glue completely dries, apply dental cement to cover the whole area and build four 'walls'

surrounding two lenses (S4H Fig).

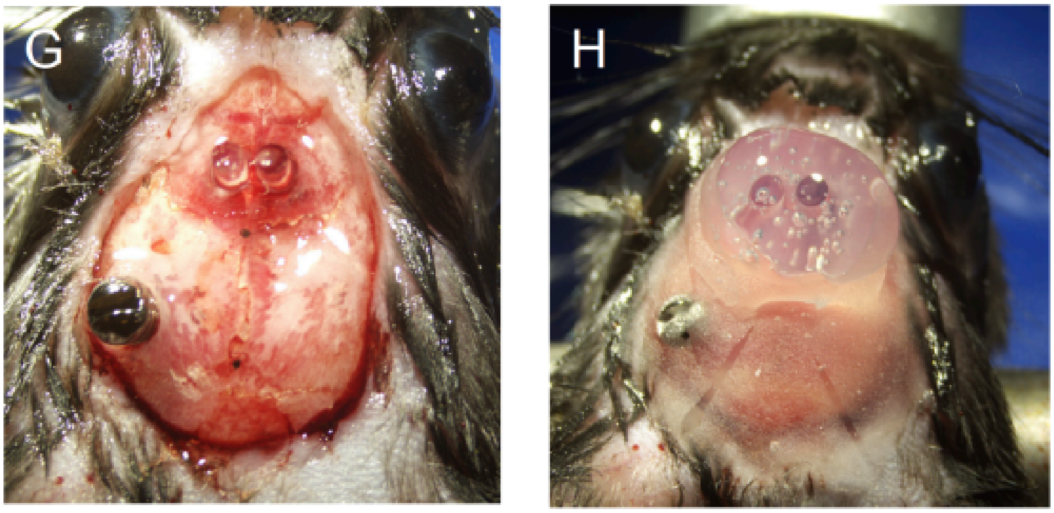

S4 Fig Implant lenses in bilateral mPFCs (G, H) Protection of implanted lenses.

53 Apply Kwik Sil on top of both lenses (S4H Fig).

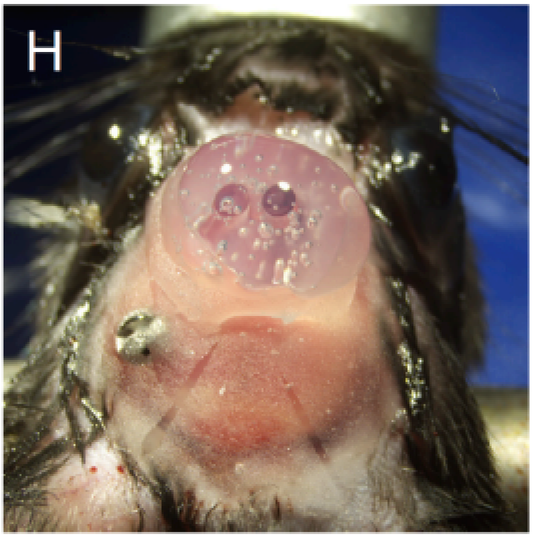

S4 Fig Implant lenses in bilateral mPFCs (G, H) Protection of implanted lenses.

54 Monitor the mouse during recovery on the heating pad and if doing well transport it back into the vivarium.

55 Complete seven days of post-operative care by performing daily subcutaneous injection 5 µL Carprofen and 0.2 µL Dexamethasone.

Part 2 Two lenses implantation: Two-lens implantation into the mPFC and NAc

2m

56 Repeat steps described in step 35 to 42 except drilling two holes and performing viral injections into these two sites (S5A Fig).

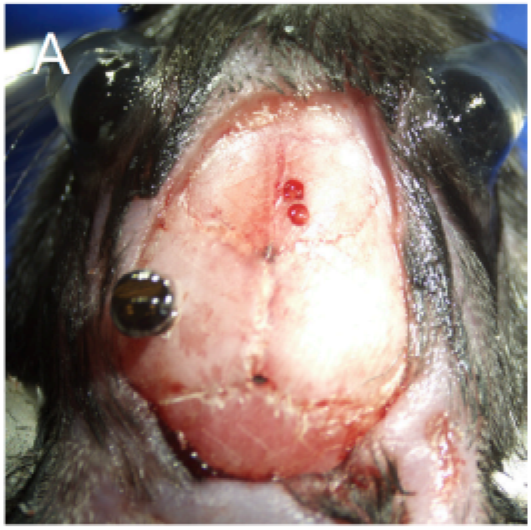

S5 Fig Dual lens implantations in ipsilateral mPFC (PL) and NAc. (A) Skull preparation

57 Move a flattened needle to bregma and measure the height. Move the needle to x,y coordinates of lens center for NAc. Lower it to the height of bregma skull top and set zero. Insert the needle into the hole and lower it to 200-300 µm above where the lens will be implanted (S5B Fig). Retract the needle and clean the top of the cavity.

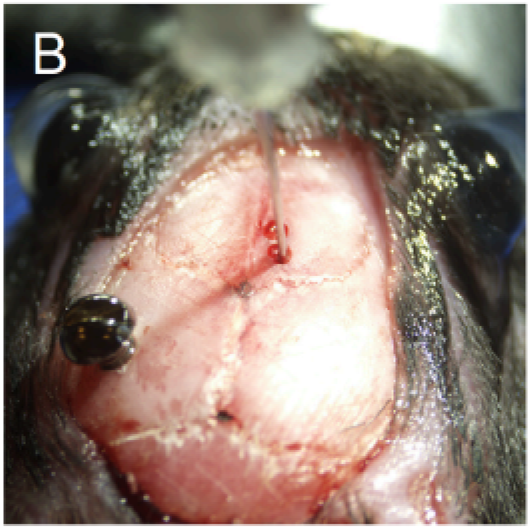

S5 Fig Dual lens implantations in ipsilateral mPFC (PL) and NAc. (B-D) NAc lens implantation.

58 Wait for 00:01:00 - 00:02:00 to make sure there is no more bleeding. During this time, prepare the lens holder and invert microscope and adjust it to a good angle to monitor the bottom of the lens. 2m

Note

In this case, because two lenses will be very close to each other, once one lens is fixed, the space around for the other lens holder will be limited. We suggest implanting the longer lens first using commercial lens holder (see Materials, Lens holder). Then implant the other one using a homemade lens holder (see Materials, Lens holder) (Lens holder pipette tip wall can be thinned to fit into the space).

59 Move the held lens to the cavity and make the bottom of the lens and the edge of the hole at the same level to set z-position zero. Lower the lens quickly to prevent bleeding (S5C Fig).

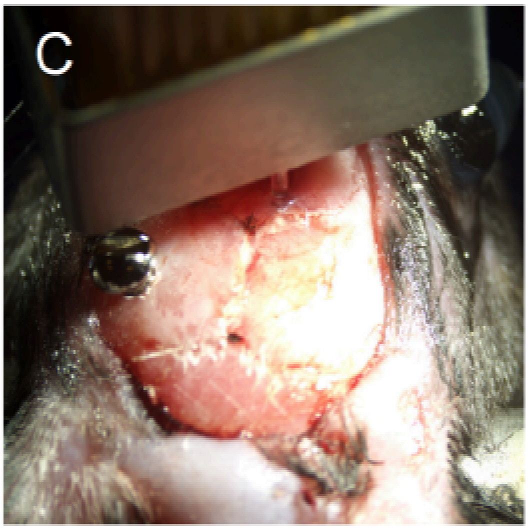

S5 Fig Dual lens implantations in ipsilateral mPFC (PL) and NAc. (B-D) NAc lens implantation.

60 Apply a small amount of cyanoacrylate glue around the bottom of the lens (S5C Fig). Apply one drop of activator to dry the glue immediately. Remove the lens holder carefully. Apply a small amount of dental cement around the bottom of the lens on the skull (S5D Fig). Be careful not to get glue or dental cement on the other hole.

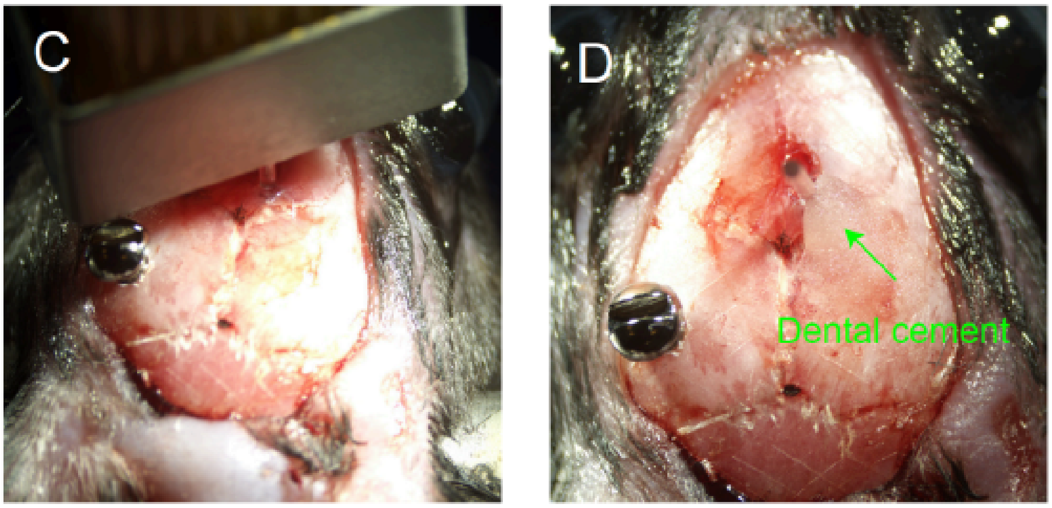

S5 Fig Dual lens implantations in ipsilateral mPFC (PL) and NAc. (B-D) NAc lens implantation.

61 Repeat step 57-60 for PFC lens implantation (S5E and S5F Fig).

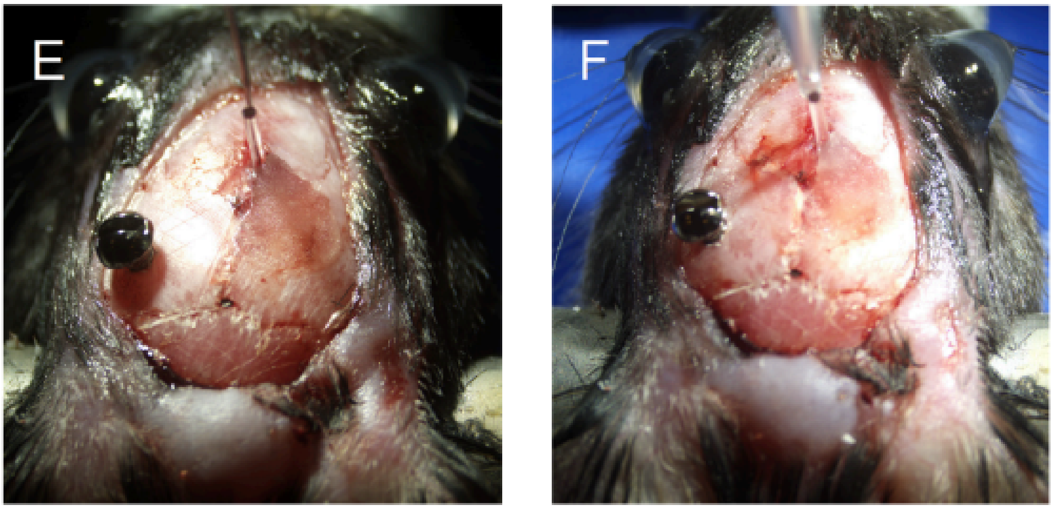

S5 Fig Dual lens implantations in ipsilateral mPFC (PL) and NAc. (E and F) mPFC lens implantation

62 Apply cyanoacrylate glue and dental cement on the skull. Apply Kwik Sil on top of both lenses (S5G and S5H Fig).

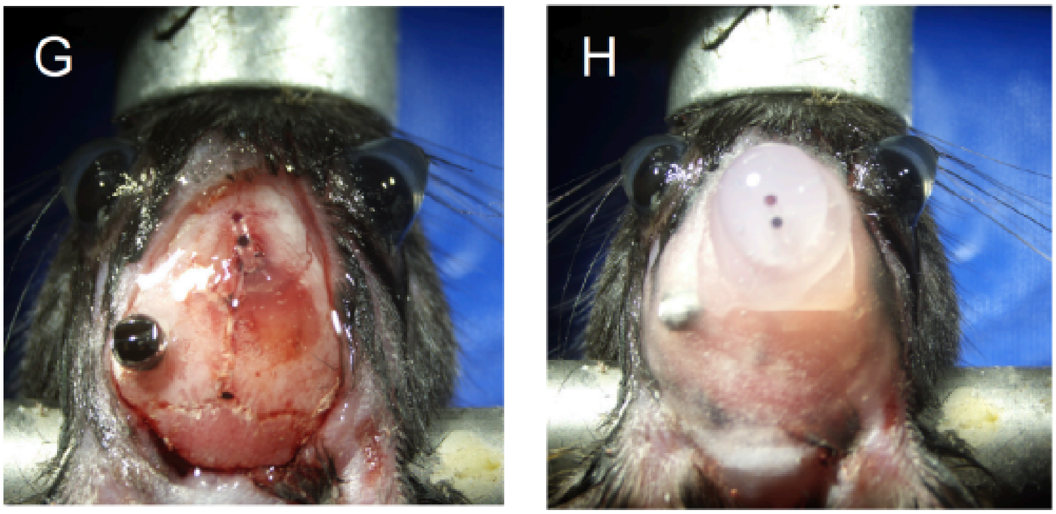

S5 Fig Dual lens implantations in ipsilateral mPFC (PL) and NAc. (G-H). Protection of implanted lenses.

63 Inject 5 µL Carprofen, 0.2 µL Dexamethasone and 0.5 mL Saline subcutaneously. Add 0.25 µL amoxicillin into water.

64 Monitor the mouse for recovery on heat pad and transport to the vivarium.

65 Seven days post-operation care with subcutaneous injection 5 µL Carprofen and 0.2 µL Dexamethasone.
