# Supplementary figures and images for: GRIN lens implantation strategies for in vivo calcium imaging using miniature microscopy
Source: PLoS One. 2025 May 12;20(5):e0323256. doi: 10.1371/journal.pone.0323256 (PMC12068630; doi:10.1371/journal.pone.0323256)

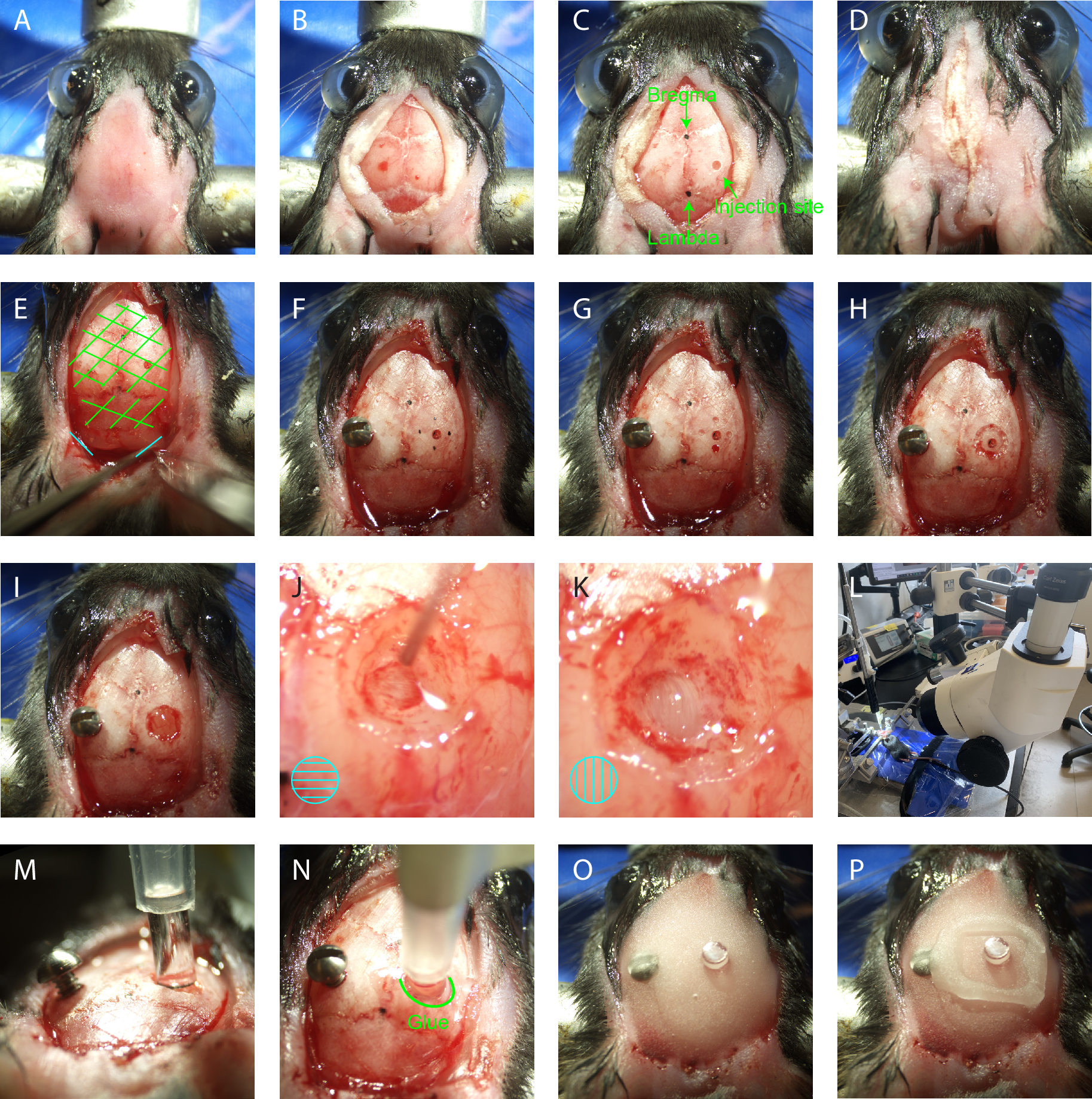

Supplement: S1 Fig — (A-D) Virus injection. (E-H) Skull preparation. (I-K) Aspiration procedure. (L-N) Lens implantation. (O, P) Protection of implanted lens. (TIF) [file pone.0323256.s002.tif]

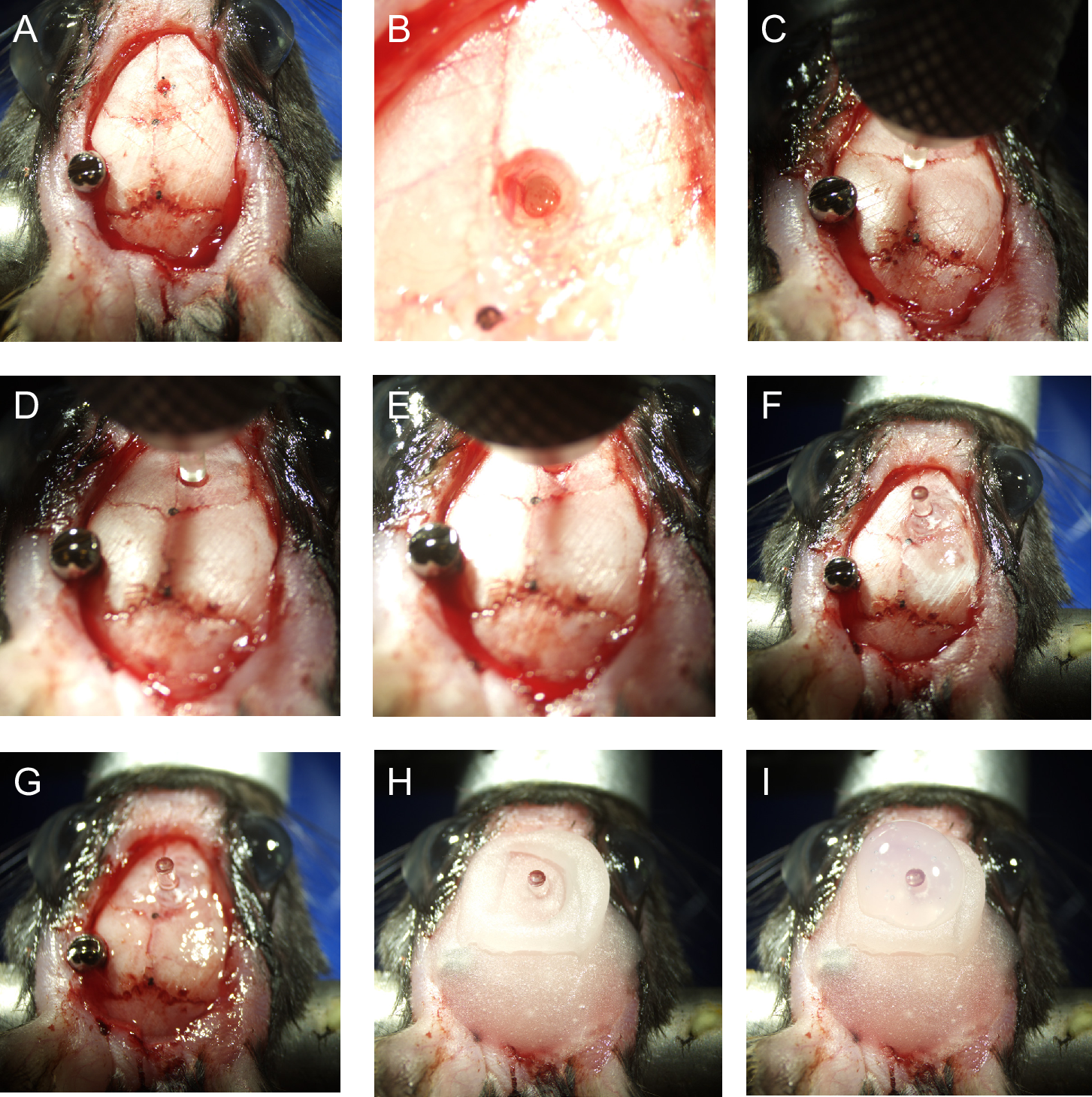

Supplement: S2 Fig — (A) Skull preparation. (B) Aspiration procedure. (C-F) Lens implantation. (G-I) Protection of implanted lens. (TIF) [file pone.0323256.s003.tif]

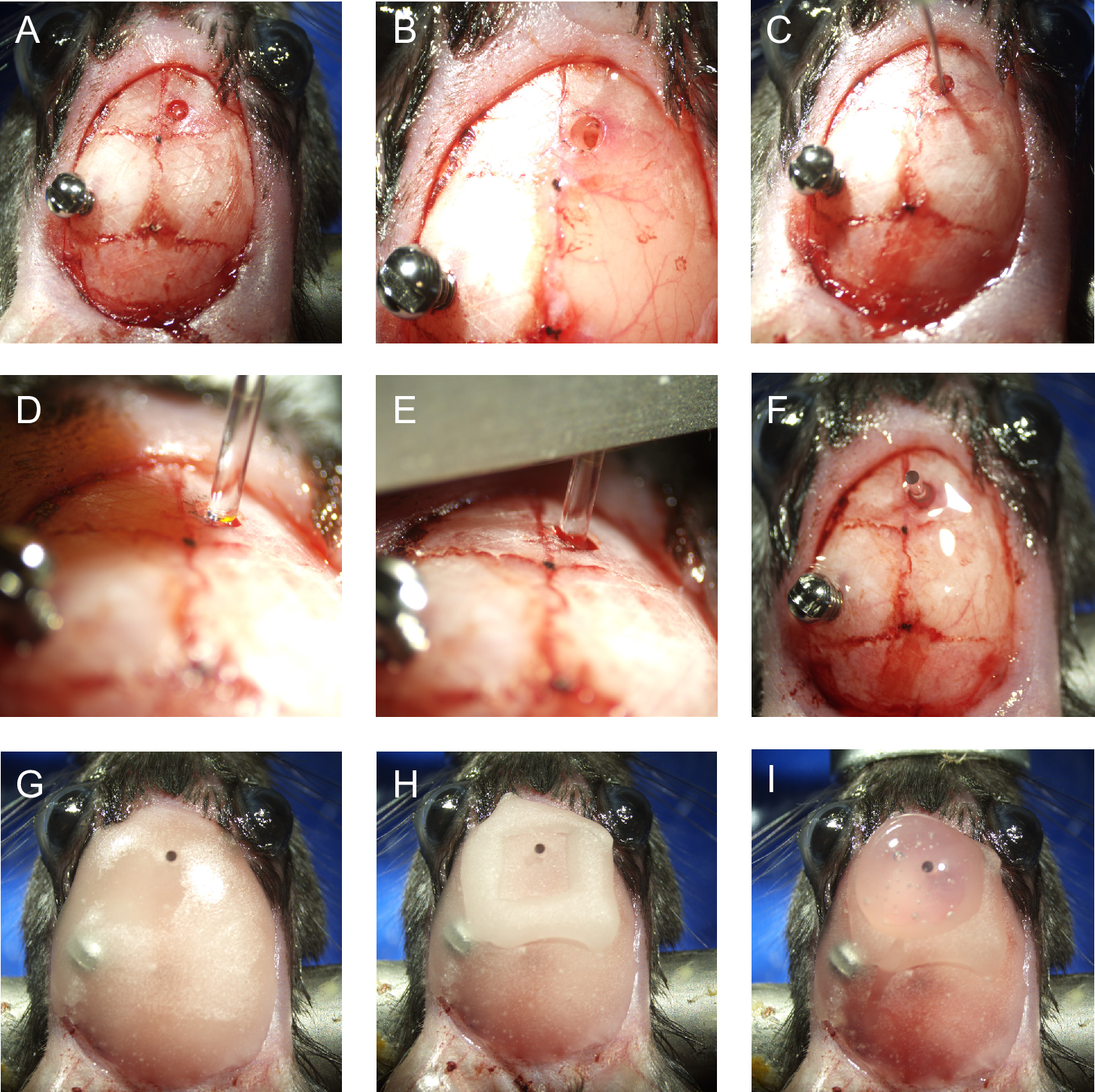

Supplement: S3 Fig — (A) Skull preparation. (B) Partial aspiration. (C) Insert a flattened needle to make a track for lens. (D,E) Lens implantation. (F-I) Protection of implanted lens. (TIF) [file pone.0323256.s004.tif]

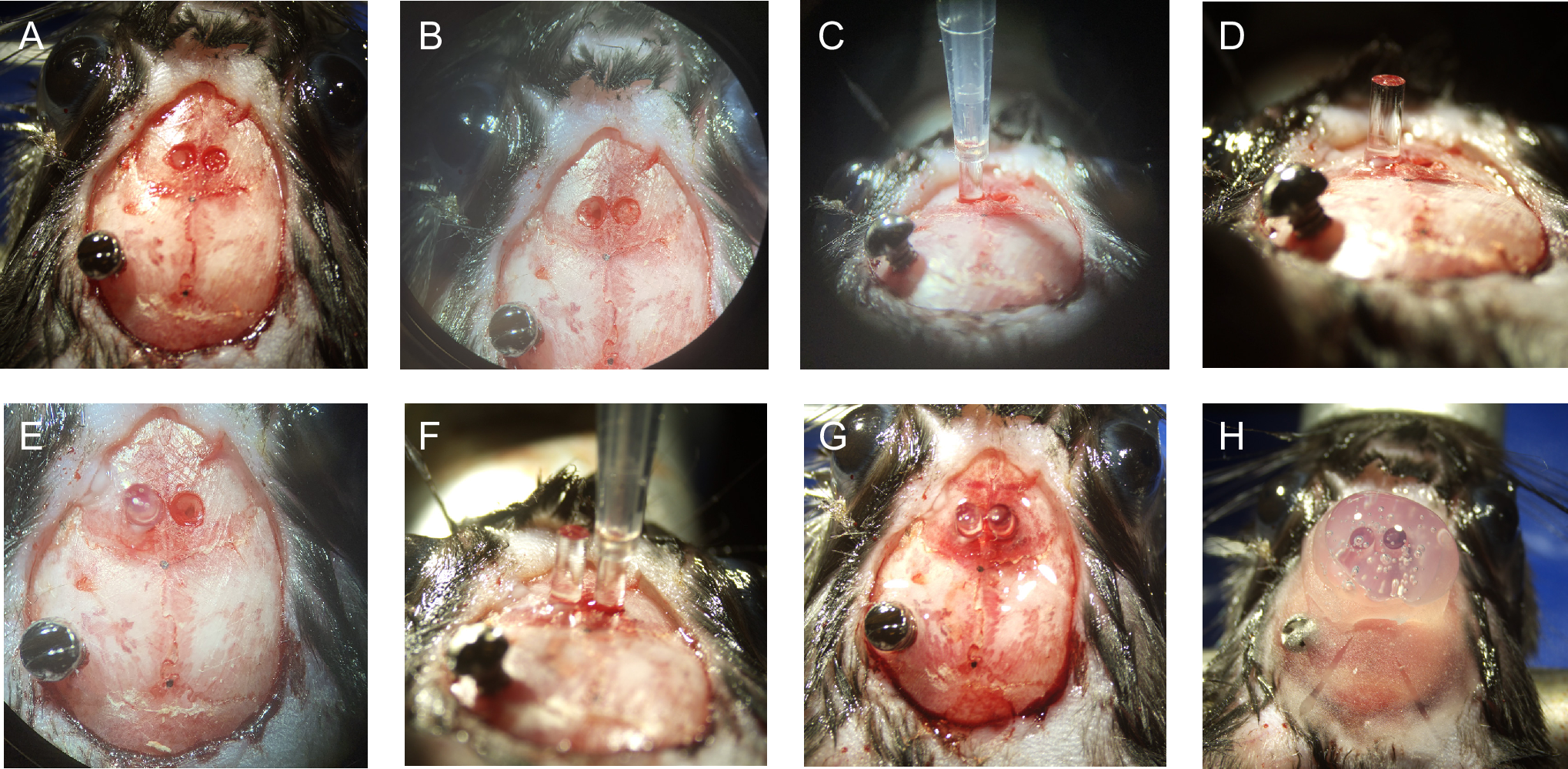

Supplement: S4 Fig — (A) Skull preparation. (B) Left mPFC aspiration. (C, D) mPFC lens implantation in left hemisphere. (E) Right mPFC aspiration. (F) mPFC lens implantation in right hemisphere. (G, H) Protection of implanted lenses. (TIF) [file pone.0323256.s005.tif]

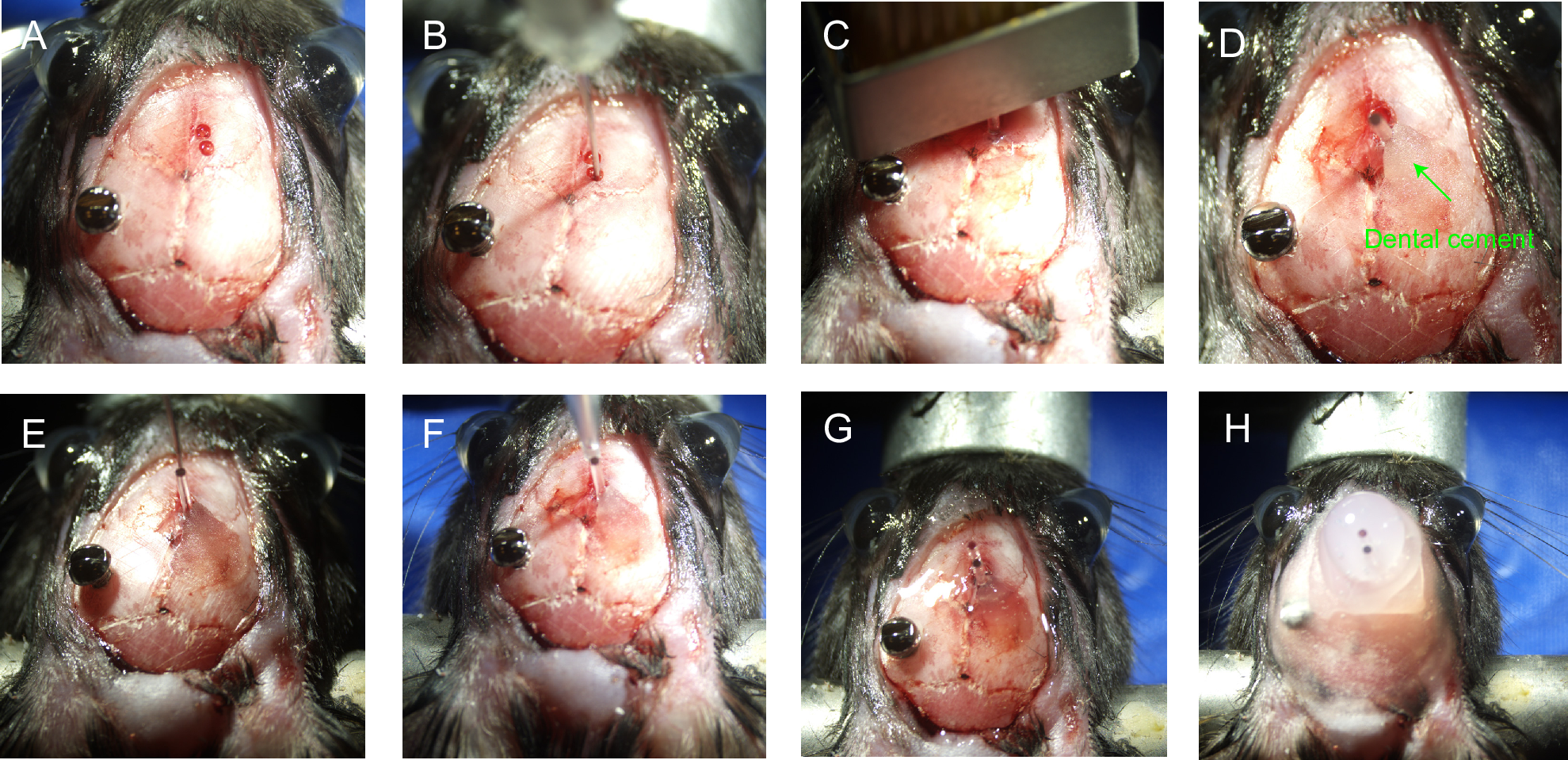

Supplement: S5 Fig — (A) Skull preparation. (B-D) NAc lens implantation. (E and F) mPFC lens implantation. (G-H). Protection of implanted lenses. (TIF) [file pone.0323256.s006.tif]

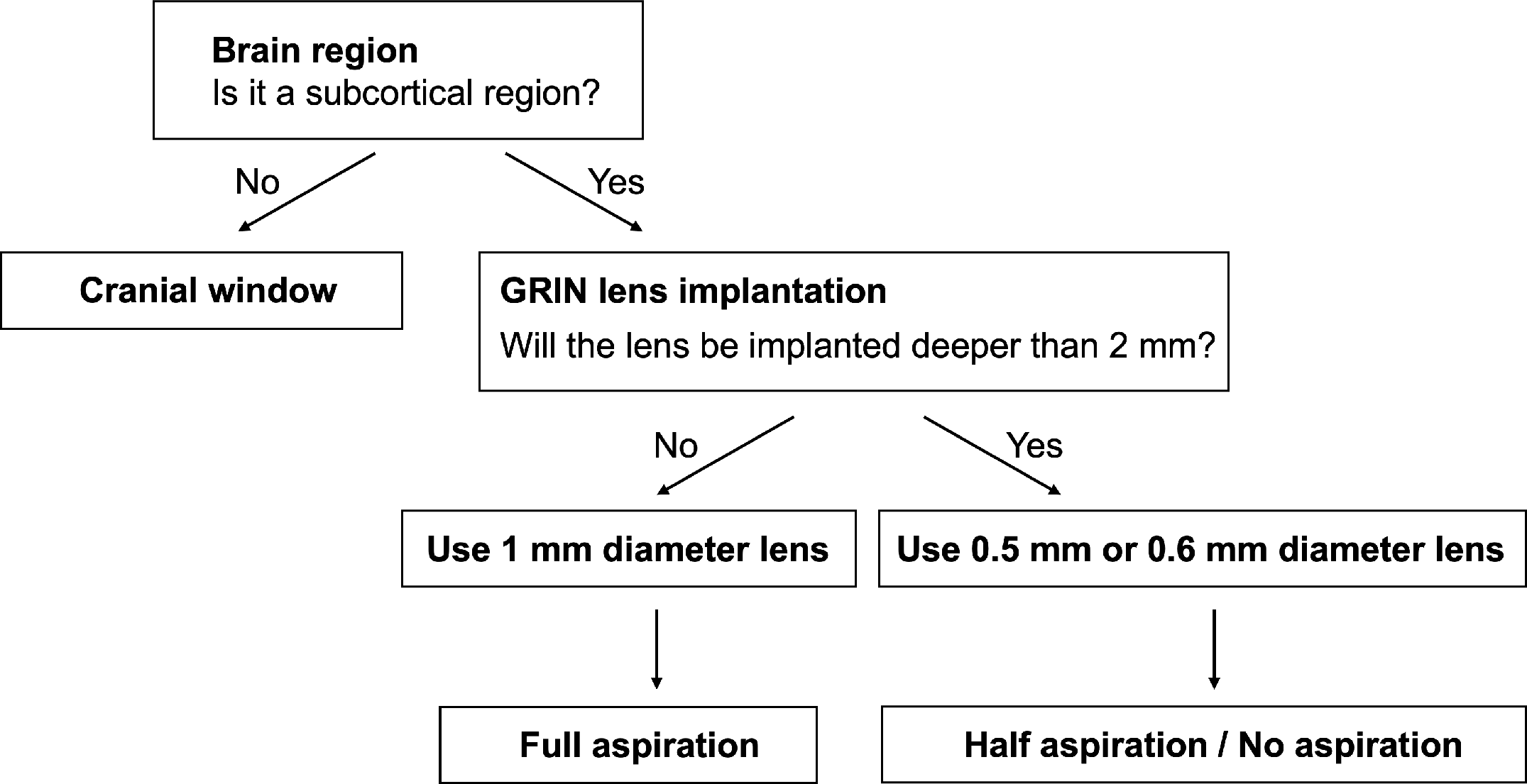

Supplement: S6 Fig — (TIF) [file pone.0323256.s007.tif]
